# Supplementary material for: Robustly federated learning model for identifying high-risk patients with postoperative gastric cancer recurrence
Source: Nat Commun. 2024 Jan 25;15:742. doi: 10.1038/s41467-024-44946-4 (PMC10811238; doi:10.1038/s41467-024-44946-4)
Supplement: Supplementary file 1 — Supplementary Information [file 41467_2024_44946_MOESM1_ESM.pdf]

Supplemental Materials

Supplementary S1: Data preprocessing

The preprocessing steps for CT images are depicted in **Figure S8**. Firstly, a doctor identifies the rectangular region of interest (ROI) within the original CT images from four data centres. The ROI is carefully selected to encompass the entire lesion's contour. Subsequently, the ROI images are resized to a rectangular shape of 224×224. Finally, each data centre contributes a varying number of ROIs, specifically 3030, 2412, 2416, and 235, respectively, for training the local model.

Supplementary S2: Federal cross-correlation information learning

The objective of federated cross-learning is to improve the generalization capability among models by learning domain-specific information across different data domains. In this study, while ensuring data security through WGAN networks, a publicly available dataset is constructed to obtain a federated co-relation matrix containing inter-domain information. Additionally, random perturbation matrices are incorporated into the generator's output to introduce random differences between the generated images and the original image data, further enhancing the security of local data.

Training of the robust local data WGAN models: In this study, a penalty term is added to the GAN network based on Wasserstein distance as the cost function, replacing the Jensen-Shannon (JS) divergence. This penalty term helps enforce parameter constraints. The structure of the WGAN model is illustrated in Figure S9 A. The loss function of the WGAN model can be represented as:

$$L(G_i, D_i) = \min_{G_i} \max_{D_i} \{ \underset{P^i_{real}}{E}[D_i(x_i)] - \underset{P^i_{fake}}{E}[D_i(G_i(z_i))] + \lambda \underset{D^i_w}{E}[\| \nabla D^i_w \|_p - 1]^2 \} \quad (2)$$

In the above equation, Let the data from the i-th centre be denoted as x_i, and its corresponding true distribution as P^i_{real}. z_i represents a tensor of data with Gaussian noise. G_i and D_i are the generator and discriminator, respectively, for the i-th centre. $\|\cdot\|_p$ denotes the P-norm. ∇ represents the gradient operator. λ is the penalty coefficient. $D^i_w = \xi x_i + (1 - G_i(z_i))$, ξ follow a uniform distribution within the range [0, 1]. P^i_{real} represents the distribution of the generated data by $G_i(z_i)$ .The loss function for G_i in the model can be defined as $L_{G_i} = 1 - D_i(G_i(z_i))$.

During the training process of G_i and D_i, the Adam optimizer is employed to

iteratively update the parameters of $L(G_i, D_i)$ and L_{G_i} by cross-interpolating G_i and D_i. It is crucial to consider that the generated data by G_i approximates the distribution of real data, which may lead to potential data leakage issues. Therefore, in this paper, Gaussian noise N is incorporated as perturbation in the output section of the original generator G_i to ensure the security of local data. The generated data D^i_{fake} for the i-th centre is represented as follows:

$$D^i_{fake} = G_i(z_i) + \alpha N \tag{3}$$

Where: α represents the perturbation intensity coefficient, N denotes the Gaussian noise that follows the distribution of $\mu = 0$ and $\sigma^2 = 1$. Subsequently, each centre model utilizes the trained G_i to generate data D^i_{fake} specific to that centre and then transmits it to the central model. The central model collects the complete set of generated data from all the centres, denoted as $D_{com} = \{D^1_{fake}, D^2_{fake}, ..., D^3_{fake}\}$. In this set, n represents the number of data centres, $\{x^i_{fake}\}$ represents the image data, and $\{y^i_{fake}\}$, $(\{x^i_{fake}\}, \{y^i_{fake}\}) \in D^i_{fake}$ represents the image labels.

Cross-federated Learning with Multi-Centre Generated Data: The goal of federated learning is to achieve consensus among different centre models for the same categories. In this paper, a cross-correlation matrix is constructed to express the inter-domain information between different local models on the same dataset, incorporating multi-centre model information. This cross-correlation matrix is defined as follows:

$$M^t = \begin{vmatrix} C^t_1(x^1_{com}) & \ldots & \ldots & C^t_1(x^k_{com}) \\ \ldots & \ldots & & \ldots \\ \ldots & & \ldots & \ldots \\ C^t_n(x^1_{com}) & \ldots & \ldots & C^t_n(x^k_{com}) \end{vmatrix} \tag{4}$$

Where: M^t represents the cross-correlation matrix for the t-th round of model training. C_i^t denotes the local model of the i-th data centre in the t-th round. x_{com}^i represents the i-th data from the Representative data set D_{com}. K represents the number of samples in the Representative data set. The cross-correlation matrix constructed in equation (4) has rows that represent the predicted logits generated by each individual local model for every data sample in the Representative data set. The matrix represents the predicted logits generated by different local models from various centres for the same data sample in the Representative data set. Therefore, this matrix provides insights into the preference level of different local models for the same data. The process of generating the cross-correlation matrix is illustrated in **Figure S9 b.**

Supplementary S3: Federated adaptive features Learning based on GCN

The goal of federated robust learning based on Graph Convolutional Networks (GCN) is to utilize a GCN network to learn the adjacency matrix $A \in \{0,1\}^{N \times N}$, which reflects the topological relationships and inter-domain information among different centres. By leveraging the adjacency matrix A, the global model can be robust, thereby enhancing the performance of local models on their respective local data.

$$\min_{\{\upsilon_1 \dots \upsilon_n\}} \sum_{i=1}^{n} (F_i(\upsilon_i) + \lambda[R(\upsilon_i, \omega) + R(\upsilon_i, u_i)]) + \gamma C(M) \tag{4}$$

where: $F(.)$ represents the local model's local loss, υ_i and u_i are the robust model parameters and local model parameters of the i-th centre, respectively, ω represents the global model parameters, λ and γ are two hyperparameters, $R(.)$ calculates the Euclidean distance between the two sets of parameters, $C(.)$ denotes the GCN network structure, and M represents the cross-correlation matrix.

Federated robust **based on GCN:** In order to incorporate inter-domain information between different models into the adjacency matrix A_i, its construction should reference the cross-correlation matrix M^t as the learning objective. Therefore, the adjacency matrix A_i is learned through the GCN network to capture the inter-domain information between M^t and the topological structure of data from different centres. Subsequently, the global model is Robust. This process can be represented as follows:

$$P_{i,j}^{new} = \sum_{i=1}^{n} \sum_{j=1}^{k} A_{i,j} P_{i,j}^{old} \tag{5}$$

Where: $P_{i,j}^{old}$ is the parameter matrix constructed by all local models, with each row representing the parameters of the i-th centre's models. n represents the number of

clients, and k represents the number of parameters in each local model. $P_{i,j}^{new}$ is the matrix of robust parameters for all models. The adjacency matrix is denoted as $A = C(M)$. Therefore, the robust model parameters for the i-th centre are represented as $u_i = P_i^{new}$.

To optimize the solution in Equation (5), we follow the following steps: First, we solve the local model loss $F(.)$ to update the local model parameters υ_i and compute the two regularization terms. The Euclidean distance between the local model and the global model, $R(\upsilon_i, \omega)$, and the Euclidean distance between the robust model and the local model, $R(u_i, v_i)$, are utilized to constrain the distances between the local model and the global model, as well as between the robust model and the local model. Afterwards, the central model aggregates the parameters of each local model to obtain the global model parameters ω. Finally, the GCN network automatically updates the model parameters u_i of each node by aggregating the inter-domain information from various centres in the graph. The robust structure is illustrated in **Figure S10**.

Algorithm 1. Federated robust based on GCN

Initialize λ, γ, η, S, T, C, A, υ_i

for each communication round s=0, 1, …, S do

    Local Update

    for local epoch t=0, 1, …, T do

        $\upsilon_i^{t+1} \leftarrow \upsilon_i^t - \eta \nabla (F_i(\upsilon_i^t) + \lambda [R(\upsilon_i^t, \omega^t) + R(\upsilon_i^t, u_i^t)])$

        end for

    Global Update

$$\omega^t \leftarrow \frac{\sum_{i=1}^{n} \upsilon_i}{n}$$

$$P_{i,j}^{s} \leftarrow Splice(\upsilon_1, ..., \upsilon_n)$$

<u>Update of adjacency matrix A</u>

$$A_{i,j} = \sum_{i=1}^{n} \sum_{j=1}^{h} Y(\upsilon_i, x_j^{fake})$$

<u>GCN Update</u>

for GCN epoch c=0, 1, …, *C* do

$$A^{c+1} \leftarrow A^{c} - \frac{1}{n} \sum_{i=1}^{n} (A^{c} - M^{c})$$

end for

<u>Robust of model</u>

$$P_{i,j}^{s+1} \leftarrow \sum_{i=1}^{n} \sum_{j=1}^{k} A_{i,j}^{s} P_{i,j}^{s}$$

$$u^{s+1} \leftarrow P_i^{s}$$

end for

The pseudocode for the parameter interaction between centres and the robust component is shown below: where n and j represent the optimization constraints; *C* is the number of GCN iterations; *T* is the number of local model training iterations; *A* is the adjacency matrix; *M* is the cross-correlation matrix learned by the local model from the representative dataset; *n* is the number of centres; *Y* is the model structure; and x represents the x_j^{fake} image in the data.

Supplementary S4: Common and adaptive features of federal radiomics

The analysis of common features and adaptive features in federated radiomics is conducted in two steps, as shown in **Figure S11**. Firstly, the robust models from each centre extract features from the test dataset of all centres and perform feature selection to eliminate the interference of model differences on the results of common and adaptive features. Subsequently, feature selection is applied to the extracted features to identify 200 federated radiomics features. Finally, the 200 radiomic features were divided into two groups to eliminate differences between categories. Subsequently, a correlation analysis was conducted on the radiomic features for recurrent advanced gastric cancer (R-AGC) and no-recurrent advanced gastric cancer (NR-AGC), and a correlation heatmap was constructed. The features with the highest correlation within the same centre and the lowest correlation across different data centres are identified as adaptive features, while the features with the highest correlation across different data centres are considered common features.

Supplementary S5: Construction of clinical models

In this study, three clinical indicators of T stage, N stage and CA199 were used to construct clinical models for the data of the four centres through the classification algorithm of decision tree.

The parameters of the decision tree classification algorithm are as follows: Mean square error as criterion term; Three fold cross verification; The tree depth is selected between 2 and 10 using the grid shrinking strategy; Splitter select the best mode;

Supplementary S6: Subjective CT findings and pathological evaluations were conducted

Image evaluations were executed by two radiologists with 10 and 15 years of experience in abdominal imaging diagnosis, respectively. Initially, the radiologists independently assessed the transverse, coronal, and sagittal CT images of the patients on the Picture Archiving and Communication System (PACS). During the evaluation process, they manipulated different CT window widths and window levels to amplify the visualization of various CT features, classifying any corresponding feature present at any CT location as positive. Subsequently, they compiled their findings and collaboratively reviewed cases with diverging opinions to resolve discrepancies.

In this study, the following five CT signs were evaluated: (1) Hyperenhancement of the adjacent gastric serosa (present or absent): The manifestation of a long band-like or patchy hyperenhancement around the lesion on the serosal side of the stomach. (2) Nodules or irregularities in the outer layer of the gastric wall (present or absent): The presence of nodules or irregularities in the outer layer of the gastric wall around the lesion. (3) Infiltration of the gastric fat (present or absent): Increased density of the fatty layer around the lesion, with linear and reticular structures indicating infiltration of the gastric fat. (4) Tumor necrosis (present or absent): The absence of enhancement within the lesion. (5) Necrosis of abdominal lymph nodes (present or absent): The absence of enhancement within the abdominal lymph nodes.

All surgical pathology samples were examined by a pathologist boasting 16 years of experience in gastrointestinal pathology. The samples were reclassified according to

the 8th edition of the TNM staging system by the American Joint Committee on Cancer (2016) (15). The evaluation encompassed histological grading, histological typing, T stage, N stage, TNM stage, lymphovascular invasion, and Lauren classification, among other indicators.

Table S1 Performance tables of four central data test sets for clinical models and RFLM

Method	Evaluation	Centre A	Centre B	Centre C	Centre D
Clinical model	AUC	0.563	0.602	0.547	0.476
	Sensitive	0.500 (20/40)	0.778 (21/27)	0.192 (5/26)	1.000 (11/11)
	Specificity	0.625 (45/72)	0.476 (20/42)	0.889 (24/27)	0.040 (2/50)
	Accuracy	0.580 (65/112)	0.594 (41/69)	0.547 (29/53)	0.213 (13/61)
	PPV	0.426 (20/47)	0.488 (21/43)	0.625 (5/8)	0.186 (11/59)
	NPV	0.692 (45/65)	0.769 (20/26)	0.533 (24/45)	1.000 (2/2)
RFLM	AUC	0.710	0.798	0.809	0.869
	Sensitive	0.700 (28/40)	0.482 (13/27)	0.731 (19/26)	0.909 (10/11)
	Specificity	0.542 (39/72)	0.857 (36/42)	0.778 (21/27)	0.640 (32/50)
	Accuracy	0.598 (67/112)	0.710 (49/69)	0.755 (40/53)	0.689 (42/61)
	PPV	0.459 (28/61)	0.684 (13/19)	0.760 (19/25)	0.357 (10/28)
	NPV	0.765 (39/51)	0.720 (36/50)	0.750 (21/28)	0.970 (32/33)

RFLM: robust federated learning model, AUC: area under the curve, PPV: positive predictive value, NPV: negative predictive value.

TableS2. Model evaluation improvement sheet

IDI			NRI	
Centre	Model 1 / Model 2	Clinical model	Model 1 / Model 2	Clinical model
Centre A	RFLM	0.1161 (p=0.0007)	RFLM	0.3722 (p=0.0004)
Centre B	RFLM	0.2285 (p=0.00006)	RFLM	0.7778 (p=0.0000)
Centre C	RFLM	0.2149 (p=0.0004)	RFLM	0.3803 (p=0.0310)
Centre D	RFLM	0.2535 (p=0.0007)	RFLM	0.5491 (p=0.0000)

IDI: integrated discrimination improvement. NRI: net reclassification improvement, RFLM: the Robust Federated Learning Model. Statistical test: Z-test (two-tailed). p: significance value. The Cut off in the NRI is defined as 0.2, 0.6.

Table S3. Performance of the seven models using four central datasets of patients in the relapse and nonrelapse models.

Method	Evaluation	Centre A	Centre B	Centre C	Centre D
FedAvg	AUC	0.672	0.726	0.711	0.798
	Sensitivity	0.575 (23/40)	0.778 (21/27)	0.769 (20/26)	0.818 (9/11)
	Specificity	0.667 (48/72)	0.381 (16/42)	0.556 (15/27)	0.600 (30/50)
	Accuracy	0.634 (71/112)	0.536 (37/69)	0.660 (35/53)	0.639 (39/61)
	PPV	0.489 (23/47)	0.447 (21/47)	0.625 (20/32)	0.310 (9/29)
	NPV	0.739 (48/65)	0.727 (16/22)	0.714 (15/21)	0.938 (30/32)
FedProx	AUC	0.658	0.718	0.731	0.766
	Sensitivity	0.600 (24/40)	0.481 (13/27)	0.807 (21/26)	0.727 (8/11)
	Specificity	0.611 (44/72)	0.810 (34/42)	0.519 (14/27)	0.520 (26/50)
	Accuracy	0.607 (68/112)	0.681 (47/69)	0.660 (35/53)	0.557 (34/61)
	PPV	0.462 (24/52)	0.619 (13/21)	0.618 (21/34)	0.250 (8/32)
	NPV	0.733 (44/60)	0.708 (34/48)	0.737 (14/19)	0.897 (26/29)
Moon	AUC	0.663	0.661	0.724	0.775
	Sensitivity	0.550 (22/40)	0.148 (4/27)	0.6539 (17/26)	0.909 (10/11)
	Specificity	0.681 (49/72)	0.833 (35/42)	0.704 (19/27)	0.640 (32/50)
	Accuracy	0.634 (71/112)	0.565 (39/69)	0.679 (36/53)	0.689 (42/61)
	PPV	0.489 (22/45)	0.364 (4/11)	0.680 (17/25)	0.357 (10/28)
	NPV	0.731 (49/67)	0.603 (35/58)	0.677 (19/28)	0.970 (32/33)
HarmoFL	AUC	0.684	0.723	0.707	0.773
	Sensitivity	0.650 (26/40)	0.704 (19/27)	0.577 (15/26)	0.273 (3/11)
	Specificity	0.597 (43/72)	0.691 (29/42)	0.630 (17/27)	0.940 (47/50)
	Accuracy	0.616 (69/112)	0.696 (48/69)	0.604 (32/53)	0.820 (50/61)
	PPV	0.473 (26/55)	0.594 (19/32)	0.600 (15/25)	0.500 (3/6)
	NPV	0.754 (43/57)	0.784 (29/37)	0.607 (17/28)	0.855 (47/55)
pFedMe	AUC	0.689	0.706	0.744	0.769
	Sensitivity	0.575 (23/40)	0.704 (19/27)	0.500 (13/26)	0.818 (9/11)
	Specificity	0.667 (48/72)	0.667 (28/42)	0.741 (20/27)	0.780 (39/50)
	Accuracy	0.634 (71/112)	0.681 (47/69)	0.623 (33/53)	0.787 (48/61)

	PPV	0.489 (23/47)	0.576 (19/33)	0.650 (13/20)	0.450 (9/20)
	NPV	0.739 (48/65)	0.778 (28/36)	0.606 (20/33)	0.951 (39/41)
pFedFSL	AUC	0.649	0.742	0.728	0.796
	Sensitivity	0.625 (25/40)	0.593 (16/27)	0.577 (15/26)	0.818 (9/11)
	Specificity	0.667 (48/72)	0.643 (27/42)	0.778 (21/27)	0.620 (31/50)
	Accuracy	0.652 (73/112)	0.623 (43/69)	0.679 (36/53)	0.656 (40/61)
	PPV	0.510 (25/49)	0.516 (16/31)	0.714 (15/21)	0.321 (9/28)
	NPV	0.762 (48/63)	0.711 (27/38)	0.656 (21/32)	0.939 (31/33)
RFLM	AUC	0.710	0.798	0.809	0.869
	Sensitivity	0.700 (28/40)	0.482 (13/27)	0.731 (19/26)	0.909 (10/11)
	Specificity	0.542 (39/72)	0.857 (36/42)	0.778 (21/27)	0.640 (32/50)
	Accuracy	0.598 (67/112)	0.710 (49/69)	0.755 (40/53)	0.689 (42/61)
	PPV	0.459 (28/61)	0.684 (13/19)	0.760 (19/25)	0.357 (10/28)
	NPV	0.765 (39/51)	0.720 (36/50)	0.750 (21/28)	0.970 (32/33)

AUC, area under the curve. PPV, positive predictive value. NPV, negative predictive value.

Table S4. LIDC multi-centre data distribution.

Set	Label (n)	Centre A	Centre B	Centre C	Centre D
Train	Benign lesion	153	145	182	152
	Malignant lesion	101	118	123	95
Test	Benign lesion	97	124	75	127
	Malignant lesion	72	66	49	67

Figure S1

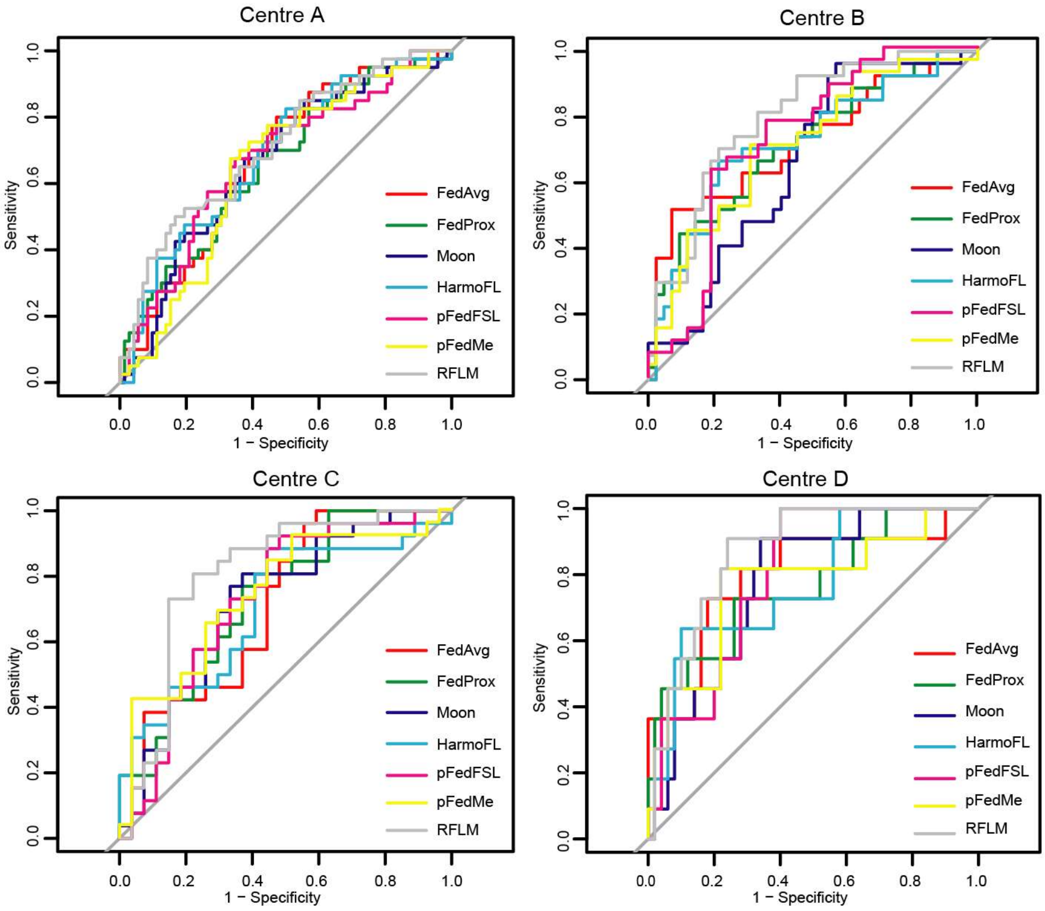

ROC curve of seven models in four data centres. Notes: RFLM: robust federated learning model.

Figure S2

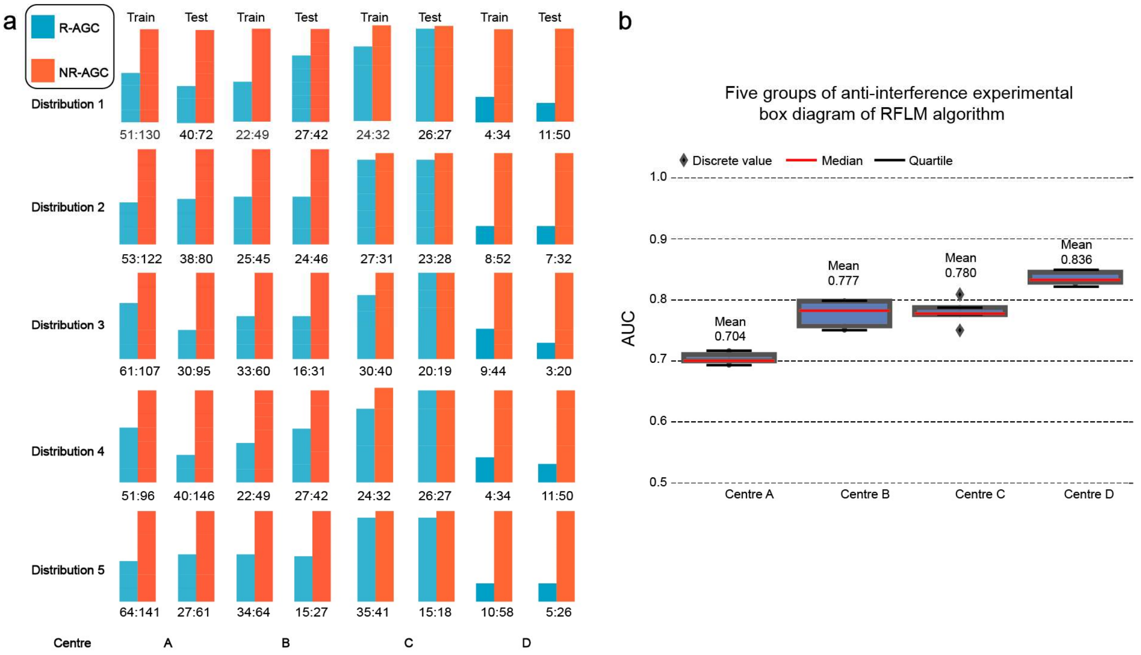

RFLM Federated Robustness Experiment. **a.** The ratios of positive and negative samples were evaluated in five experiments. **b.** Boxplots were created to visualize the results of the five RFLM robustness experiments. The upper and lower black lines in the boxplots indicate the 75th percentile values. Note: RFLM refers to the Robust Federated Learning Model, NR-AGC represents no-recurrent advanced gastric cancer, and R-AGC represents recurrent advanced gastric cancer.

Figure S3

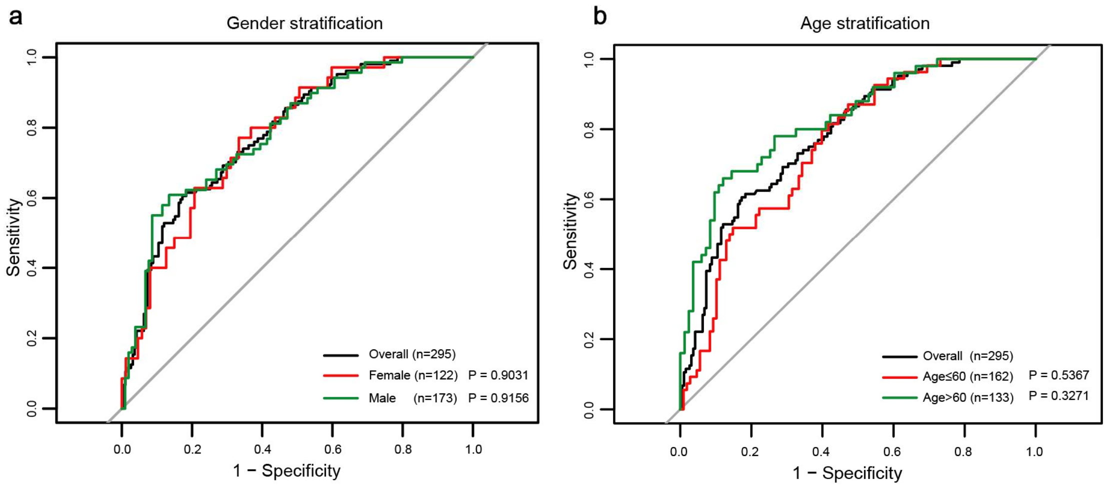

Stratified analysis results based on gender and age. **a.** Gender stratification. **b.** Age stratification. Notes: Statistical test: Delong test (two-tailed). P: significance value.

Figure S4

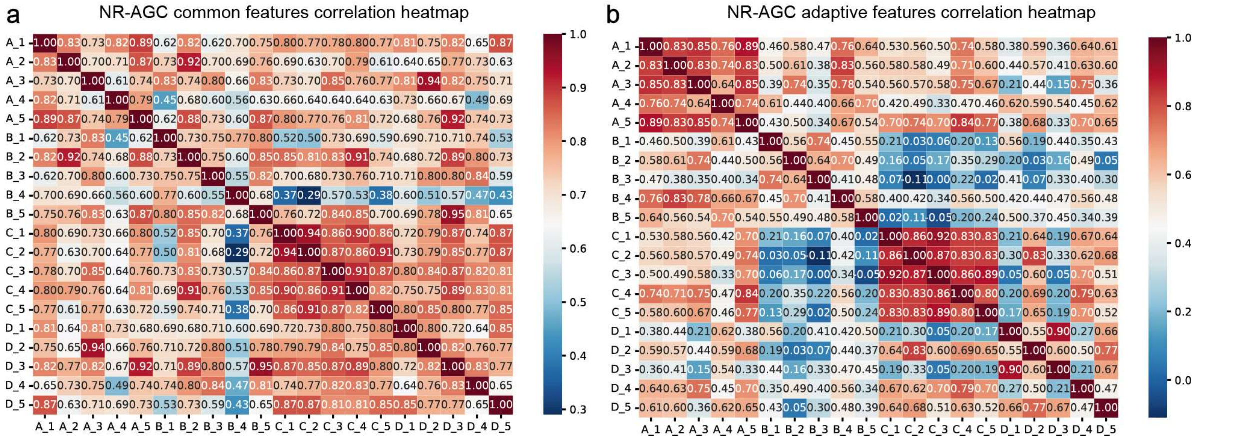

Correlation heatmap between common and adaptive features in the NR-AGC. **a.** Common features correlation heatmap of NR-AGC. **b.** Adaptive features correlation heatmap of NR-AGC. Notes: A_1: the first feature under centre A; NR-AGC: no-recurrent advanced gastric cancer.

Figure S5

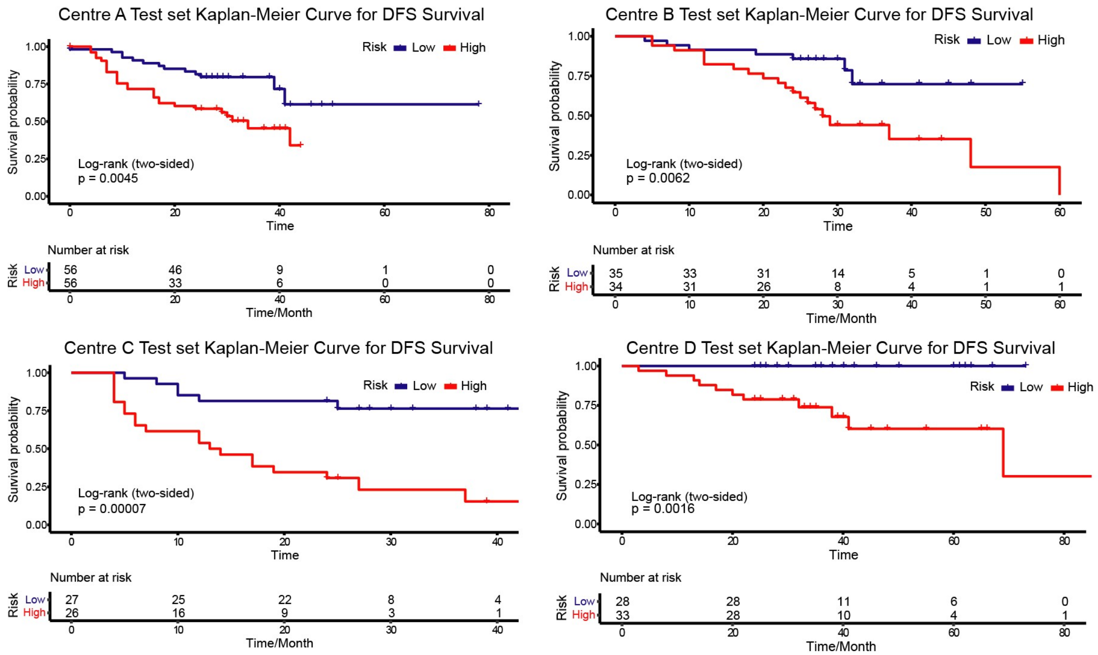

Disease free survival (DFS) analysis of four data centre test sets. Notes: Statistical test: log-rank (two-tailed),on one degrees of freedom. p: significance value.

Figure S6

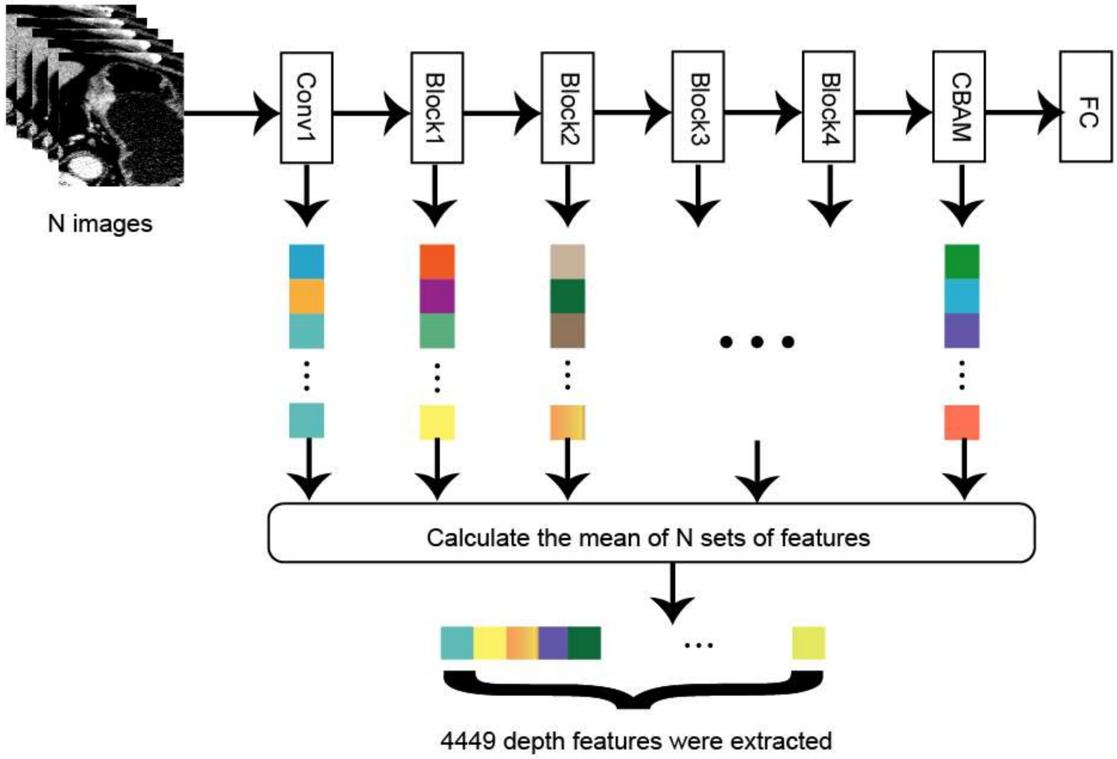

The figure represents the process of feature extraction from a patient's CT images. In this study, a ResNet18 CBAM model with spatial attention mechanism is used as the feature extraction part. Due to the presence of 4449 convolutional filters in the feature extraction network, 4449 features are extracted for each image. The features from the same filter are averaged over N instances. As a result, each patient is associated with a matrix containing 4449 features. Notes: CBAM: convolutional block attention module. Conv: convolution layer. Block1 to Block4 are the backbone of the Resnet18 network. FC: fully connected layer.

Figure S7

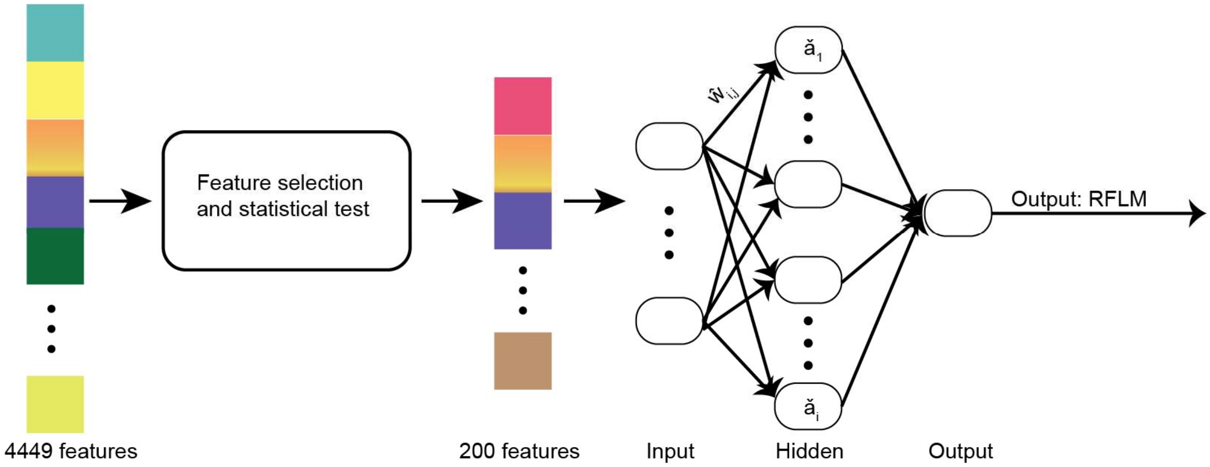

RFLM build process diagram. 4449 federated radiomics features undergo a statistical test and feature selection process, resulting in the selection of 200 federated radiomics features. These selected features are then fed into the Sparse Bayesian Extreme Learning Machine (ELM) to construct the RFLM (Robust Federated Learning Model). Notes: RFLM refers to the Robust Federated Learning System, and it encompasses the concept of federated radiomics labels. Notes: $a_i^{\vee}$ and $w_{i,j}^{\wedge}$ are the parameters and corresponding weights of ELM's hidden layer neurons, respectively

Figure S8

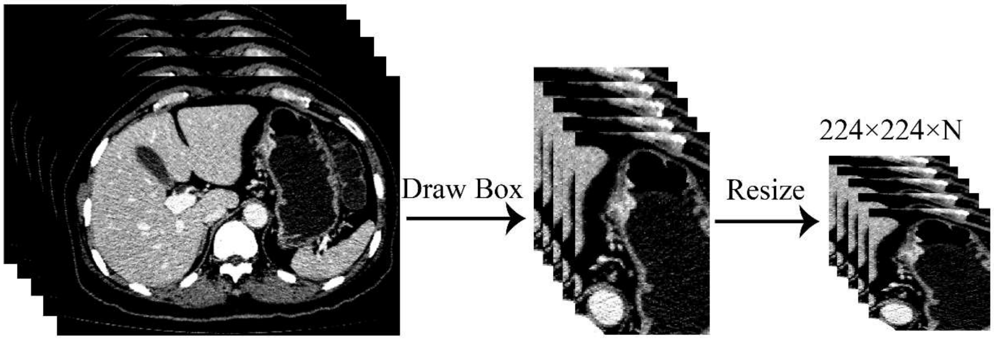

The preprocessing process of a single patient's CT images is as follows. Notes: N is the number of CT images the patient has.

Figure S9

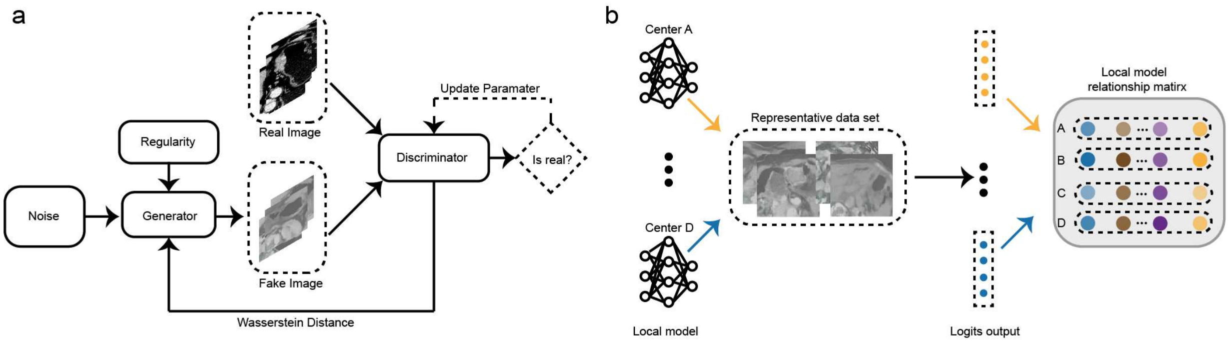

a. Schematic diagram of WGAN. **b.** The learning process of federated cross-correlation information. Representative data set: The Representative data set is constructed by each data centre using the Fake Images generated through the WGAN network. Logits out: It represents the prediction values obtained by each local model when evaluating the common data. Notes: A, B, C, and D are the names of the four centres respectively.

Figure S10

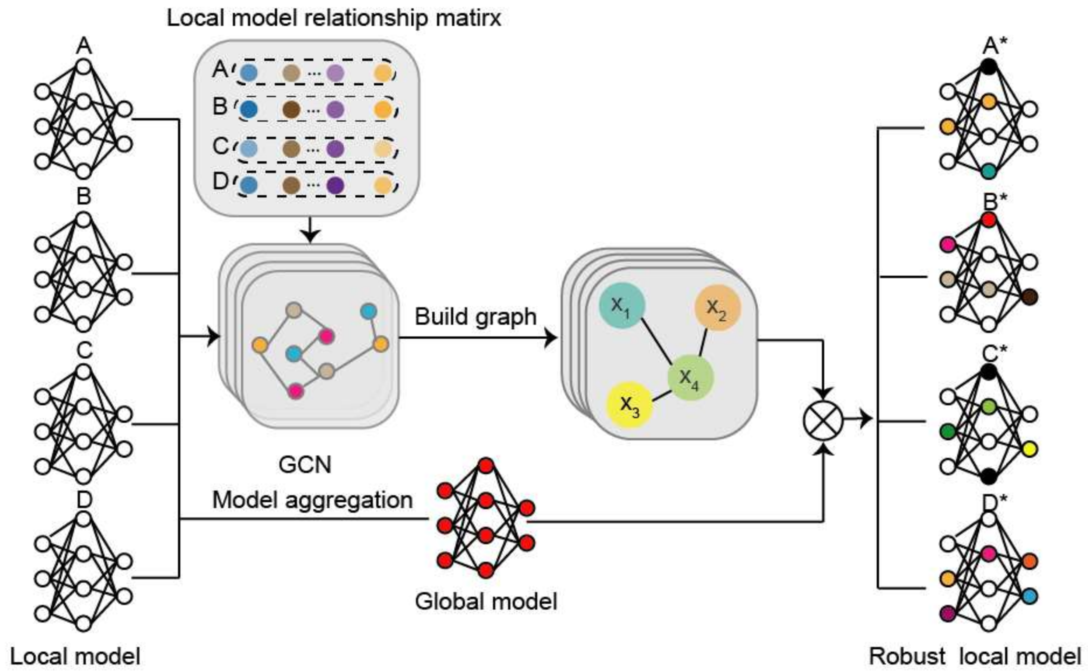

Federated robust based on GCN. Notes: GCN: Graph convolutional neural network. Adjacency matrix diagram of X1, X2, X3, and X4 data centres. A, B, C, and D are the names of the four centres respectively.

Figure S11

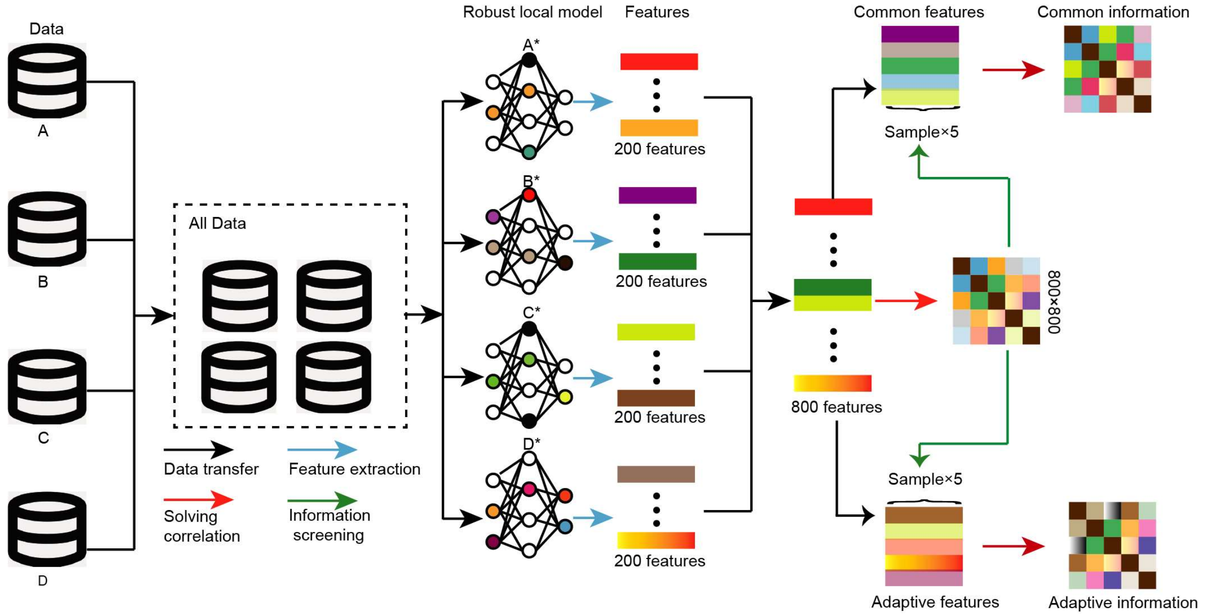

Flow chart of federal radiomics commonality and adaptive analysis. Notes: A, B, C, and D are the names of the four centres respectively. A*, B*, C*, and D* are robust local models of these four centres, respectively.
