# Supplementary material for: Robustly federated learning model for identifying high-risk patients with postoperative gastric cancer recurrence
Source: Nat Commun. 2024 Jan 25;15:742. doi: 10.1038/s41467-024-44946-4 (PMC10811238; doi:10.1038/s41467-024-44946-4)
Supplement: Supplementary file 3 — Description of Additional Supplementary Files [file 41467_2024_44946_MOESM3_ESM.pdf]

## Description of Additional Supplementary Files

**Supplementary Data 1:** the LIDC data used in the article.

**Supplementary Code:** The code files used in this article.
