# Supplementary figures and images for: Robustly federated learning model for identifying high-risk patients with postoperative gastric cancer recurrence (part 2 of 2)
Source: Nat Commun. 2024 Jan 25;15:742. doi: 10.1038/s41467-024-44946-4 (PMC10811238; doi:10.1038/s41467-024-44946-4)

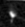

Supplement: Supplementary file 4 — Supplementary Data 1 [file 41467_2024_44946_MOESM4_ESM.zip › Supplementary Data 1/LIDC/LIDC/0/test_data/0/LIDC-IDRI-0655_1/57346_000490_01-01-2000-36060_1326-NLST TLC VOL B30F-53512_doctor_anon_session_0.png]

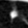

Supplement: Supplementary file 4 — Supplementary Data 1 [file 41467_2024_44946_MOESM4_ESM.zip › Supplementary Data 1/LIDC/LIDC/0/test_data/0/LIDC-IDRI-0655_1/57346_000501_01-01-2000-36060_1326-NLST TLC VOL B30F-53512_doctor_anon_session_0.png]

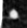

Supplement: Supplementary file 4 — Supplementary Data 1 [file 41467_2024_44946_MOESM4_ESM.zip › Supplementary Data 1/LIDC/LIDC/0/test_data/0/LIDC-IDRI-0655_2/13639_000050_01-01-2000-36060_1326-NLST TLC VOL B30F-53512_doctor_anon_session_2.png]

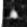

Supplement: Supplementary file 4 — Supplementary Data 1 [file 41467_2024_44946_MOESM4_ESM.zip › Supplementary Data 1/LIDC/LIDC/0/test_data/0/LIDC-IDRI-0655_2/13639_000095_01-01-2000-36060_1326-NLST TLC VOL B30F-53512_doctor_anon_session_2.png]

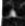

Supplement: Supplementary file 4 — Supplementary Data 1 [file 41467_2024_44946_MOESM4_ESM.zip › Supplementary Data 1/LIDC/LIDC/0/test_data/0/LIDC-IDRI-0655_2/13639_000096_01-01-2000-36060_1326-NLST TLC VOL B30F-53512_doctor_anon_session_2.png]

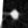

Supplement: Supplementary file 4 — Supplementary Data 1 [file 41467_2024_44946_MOESM4_ESM.zip › Supplementary Data 1/LIDC/LIDC/0/test_data/0/LIDC-IDRI-0655_2/13639_000265_01-01-2000-36060_1326-NLST TLC VOL B30F-53512_doctor_anon_session_2.png]

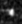

Supplement: Supplementary file 4 — Supplementary Data 1 [file 41467_2024_44946_MOESM4_ESM.zip › Supplementary Data 1/LIDC/LIDC/0/test_data/0/LIDC-IDRI-0655_2/13639_000274_01-01-2000-36060_1326-NLST TLC VOL B30F-53512_doctor_anon_session_2.png]

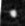

Supplement: Supplementary file 4 — Supplementary Data 1 [file 41467_2024_44946_MOESM4_ESM.zip › Supplementary Data 1/LIDC/LIDC/0/test_data/0/LIDC-IDRI-0655_2/13639_000426_01-01-2000-36060_1326-NLST TLC VOL B30F-53512_doctor_anon_session_2.png]

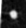

Supplement: Supplementary file 4 — Supplementary Data 1 [file 41467_2024_44946_MOESM4_ESM.zip › Supplementary Data 1/LIDC/LIDC/0/test_data/0/LIDC-IDRI-0655_2/13639_000430_01-01-2000-36060_1326-NLST TLC VOL B30F-53512_doctor_anon_session_2.png]

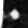

Supplement: Supplementary file 4 — Supplementary Data 1 [file 41467_2024_44946_MOESM4_ESM.zip › Supplementary Data 1/LIDC/LIDC/0/test_data/0/LIDC-IDRI-0655_2/13639_000456_01-01-2000-36060_1326-NLST TLC VOL B30F-53512_doctor_anon_session_2.png]

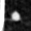

Supplement: Supplementary file 4 — Supplementary Data 1 [file 41467_2024_44946_MOESM4_ESM.zip › Supplementary Data 1/LIDC/LIDC/0/test_data/0/LIDC-IDRI-0655_2/57338_000050_01-01-2000-36060_1326-NLST TLC VOL B30F-53512_doctor_anon_session_0.png]

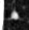

Supplement: Supplementary file 4 — Supplementary Data 1 [file 41467_2024_44946_MOESM4_ESM.zip › Supplementary Data 1/LIDC/LIDC/0/test_data/0/LIDC-IDRI-0655_2/57338_000095_01-01-2000-36060_1326-NLST TLC VOL B30F-53512_doctor_anon_session_0.png]

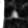

Supplement: Supplementary file 4 — Supplementary Data 1 [file 41467_2024_44946_MOESM4_ESM.zip › Supplementary Data 1/LIDC/LIDC/0/test_data/0/LIDC-IDRI-0655_2/57338_000096_01-01-2000-36060_1326-NLST TLC VOL B30F-53512_doctor_anon_session_0.png]

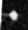

Supplement: Supplementary file 4 — Supplementary Data 1 [file 41467_2024_44946_MOESM4_ESM.zip › Supplementary Data 1/LIDC/LIDC/0/test_data/0/LIDC-IDRI-0655_2/57338_000265_01-01-2000-36060_1326-NLST TLC VOL B30F-53512_doctor_anon_session_0.png]

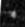

Supplement: Supplementary file 4 — Supplementary Data 1 [file 41467_2024_44946_MOESM4_ESM.zip › Supplementary Data 1/LIDC/LIDC/0/test_data/0/LIDC-IDRI-0655_2/57338_000274_01-01-2000-36060_1326-NLST TLC VOL B30F-53512_doctor_anon_session_0.png]

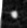

Supplement: Supplementary file 4 — Supplementary Data 1 [file 41467_2024_44946_MOESM4_ESM.zip › Supplementary Data 1/LIDC/LIDC/0/test_data/0/LIDC-IDRI-0655_2/57338_000426_01-01-2000-36060_1326-NLST TLC VOL B30F-53512_doctor_anon_session_0.png]

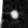

Supplement: Supplementary file 4 — Supplementary Data 1 [file 41467_2024_44946_MOESM4_ESM.zip › Supplementary Data 1/LIDC/LIDC/0/test_data/0/LIDC-IDRI-0655_2/57338_000430_01-01-2000-36060_1326-NLST TLC VOL B30F-53512_doctor_anon_session_0.png]

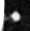

Supplement: Supplementary file 4 — Supplementary Data 1 [file 41467_2024_44946_MOESM4_ESM.zip › Supplementary Data 1/LIDC/LIDC/0/test_data/0/LIDC-IDRI-0655_2/57338_000456_01-01-2000-36060_1326-NLST TLC VOL B30F-53512_doctor_anon_session_0.png]

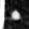

Supplement: Supplementary file 4 — Supplementary Data 1 [file 41467_2024_44946_MOESM4_ESM.zip › Supplementary Data 1/LIDC/LIDC/0/test_data/0/LIDC-IDRI-0655_2/9_000050_01-01-2000-36060_1326-NLST TLC VOL B30F-53512_doctor_anon_session_3.png]

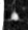

Supplement: Supplementary file 4 — Supplementary Data 1 [file 41467_2024_44946_MOESM4_ESM.zip › Supplementary Data 1/LIDC/LIDC/0/test_data/0/LIDC-IDRI-0655_2/9_000095_01-01-2000-36060_1326-NLST TLC VOL B30F-53512_doctor_anon_session_3.png]

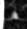

Supplement: Supplementary file 4 — Supplementary Data 1 [file 41467_2024_44946_MOESM4_ESM.zip › Supplementary Data 1/LIDC/LIDC/0/test_data/0/LIDC-IDRI-0655_2/9_000096_01-01-2000-36060_1326-NLST TLC VOL B30F-53512_doctor_anon_session_3.png]

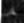

Supplement: Supplementary file 4 — Supplementary Data 1 [file 41467_2024_44946_MOESM4_ESM.zip › Supplementary Data 1/LIDC/LIDC/0/test_data/0/LIDC-IDRI-0655_2/9_000245_01-01-2000-36060_1326-NLST TLC VOL B30F-53512_doctor_anon_session_3.png]

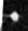

Supplement: Supplementary file 4 — Supplementary Data 1 [file 41467_2024_44946_MOESM4_ESM.zip › Supplementary Data 1/LIDC/LIDC/0/test_data/0/LIDC-IDRI-0655_2/9_000265_01-01-2000-36060_1326-NLST TLC VOL B30F-53512_doctor_anon_session_3.png]

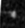

Supplement: Supplementary file 4 — Supplementary Data 1 [file 41467_2024_44946_MOESM4_ESM.zip › Supplementary Data 1/LIDC/LIDC/0/test_data/0/LIDC-IDRI-0655_2/9_000274_01-01-2000-36060_1326-NLST TLC VOL B30F-53512_doctor_anon_session_3.png]

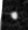

Supplement: Supplementary file 4 — Supplementary Data 1 [file 41467_2024_44946_MOESM4_ESM.zip › Supplementary Data 1/LIDC/LIDC/0/test_data/0/LIDC-IDRI-0655_2/9_000426_01-01-2000-36060_1326-NLST TLC VOL B30F-53512_doctor_anon_session_3.png]

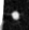

Supplement: Supplementary file 4 — Supplementary Data 1 [file 41467_2024_44946_MOESM4_ESM.zip › Supplementary Data 1/LIDC/LIDC/0/test_data/0/LIDC-IDRI-0655_2/9_000430_01-01-2000-36060_1326-NLST TLC VOL B30F-53512_doctor_anon_session_3.png]

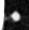

Supplement: Supplementary file 4 — Supplementary Data 1 [file 41467_2024_44946_MOESM4_ESM.zip › Supplementary Data 1/LIDC/LIDC/0/test_data/0/LIDC-IDRI-0655_2/9_000456_01-01-2000-36060_1326-NLST TLC VOL B30F-53512_doctor_anon_session_3.png]

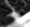

Supplement: Supplementary file 4 — Supplementary Data 1 [file 41467_2024_44946_MOESM4_ESM.zip › Supplementary Data 1/LIDC/LIDC/0/test_data/0/LIDC-IDRI-0655_3/21_000054_01-01-2000-36060_1326-NLST TLC VOL B30F-53512_doctor_anon_session_3.png]

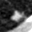

Supplement: Supplementary file 4 — Supplementary Data 1 [file 41467_2024_44946_MOESM4_ESM.zip › Supplementary Data 1/LIDC/LIDC/0/test_data/0/LIDC-IDRI-0655_3/21_000131_01-01-2000-36060_1326-NLST TLC VOL B30F-53512_doctor_anon_session_3.png]

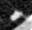

Supplement: Supplementary file 4 — Supplementary Data 1 [file 41467_2024_44946_MOESM4_ESM.zip › Supplementary Data 1/LIDC/LIDC/0/test_data/0/LIDC-IDRI-0655_3/21_000181_01-01-2000-36060_1326-NLST TLC VOL B30F-53512_doctor_anon_session_3.png]

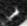

Supplement: Supplementary file 4 — Supplementary Data 1 [file 41467_2024_44946_MOESM4_ESM.zip › Supplementary Data 1/LIDC/LIDC/0/test_data/0/LIDC-IDRI-0655_3/21_000217_01-01-2000-36060_1326-NLST TLC VOL B30F-53512_doctor_anon_session_3.png]

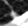

Supplement: Supplementary file 4 — Supplementary Data 1 [file 41467_2024_44946_MOESM4_ESM.zip › Supplementary Data 1/LIDC/LIDC/0/test_data/0/LIDC-IDRI-0655_3/21_000297_01-01-2000-36060_1326-NLST TLC VOL B30F-53512_doctor_anon_session_3.png]

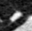

Supplement: Supplementary file 4 — Supplementary Data 1 [file 41467_2024_44946_MOESM4_ESM.zip › Supplementary Data 1/LIDC/LIDC/0/test_data/0/LIDC-IDRI-0655_3/21_000304_01-01-2000-36060_1326-NLST TLC VOL B30F-53512_doctor_anon_session_3.png]

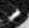

Supplement: Supplementary file 4 — Supplementary Data 1 [file 41467_2024_44946_MOESM4_ESM.zip › Supplementary Data 1/LIDC/LIDC/0/test_data/0/LIDC-IDRI-0655_3/21_000425_01-01-2000-36060_1326-NLST TLC VOL B30F-53512_doctor_anon_session_3.png]

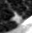

Supplement: Supplementary file 4 — Supplementary Data 1 [file 41467_2024_44946_MOESM4_ESM.zip › Supplementary Data 1/LIDC/LIDC/0/test_data/0/LIDC-IDRI-0655_3/21_000548_01-01-2000-36060_1326-NLST TLC VOL B30F-53512_doctor_anon_session_3.png]

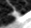

Supplement: Supplementary file 4 — Supplementary Data 1 [file 41467_2024_44946_MOESM4_ESM.zip › Supplementary Data 1/LIDC/LIDC/0/test_data/0/LIDC-IDRI-0655_3/57342_000054_01-01-2000-36060_1326-NLST TLC VOL B30F-53512_doctor_anon_session_0.png]

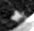

Supplement: Supplementary file 4 — Supplementary Data 1 [file 41467_2024_44946_MOESM4_ESM.zip › Supplementary Data 1/LIDC/LIDC/0/test_data/0/LIDC-IDRI-0655_3/57342_000131_01-01-2000-36060_1326-NLST TLC VOL B30F-53512_doctor_anon_session_0.png]

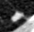

Supplement: Supplementary file 4 — Supplementary Data 1 [file 41467_2024_44946_MOESM4_ESM.zip › Supplementary Data 1/LIDC/LIDC/0/test_data/0/LIDC-IDRI-0655_3/57342_000181_01-01-2000-36060_1326-NLST TLC VOL B30F-53512_doctor_anon_session_0.png]

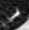

Supplement: Supplementary file 4 — Supplementary Data 1 [file 41467_2024_44946_MOESM4_ESM.zip › Supplementary Data 1/LIDC/LIDC/0/test_data/0/LIDC-IDRI-0655_3/57342_000217_01-01-2000-36060_1326-NLST TLC VOL B30F-53512_doctor_anon_session_0.png]

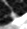

Supplement: Supplementary file 4 — Supplementary Data 1 [file 41467_2024_44946_MOESM4_ESM.zip › Supplementary Data 1/LIDC/LIDC/0/test_data/0/LIDC-IDRI-0655_3/57342_000297_01-01-2000-36060_1326-NLST TLC VOL B30F-53512_doctor_anon_session_0.png]

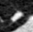

Supplement: Supplementary file 4 — Supplementary Data 1 [file 41467_2024_44946_MOESM4_ESM.zip › Supplementary Data 1/LIDC/LIDC/0/test_data/0/LIDC-IDRI-0655_3/57342_000304_01-01-2000-36060_1326-NLST TLC VOL B30F-53512_doctor_anon_session_0.png]

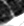

Supplement: Supplementary file 4 — Supplementary Data 1 [file 41467_2024_44946_MOESM4_ESM.zip › Supplementary Data 1/LIDC/LIDC/0/test_data/0/LIDC-IDRI-0655_3/57342_000340_01-01-2000-36060_1326-NLST TLC VOL B30F-53512_doctor_anon_session_0.png]

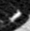

Supplement: Supplementary file 4 — Supplementary Data 1 [file 41467_2024_44946_MOESM4_ESM.zip › Supplementary Data 1/LIDC/LIDC/0/test_data/0/LIDC-IDRI-0655_3/57342_000425_01-01-2000-36060_1326-NLST TLC VOL B30F-53512_doctor_anon_session_0.png]

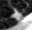

Supplement: Supplementary file 4 — Supplementary Data 1 [file 41467_2024_44946_MOESM4_ESM.zip › Supplementary Data 1/LIDC/LIDC/0/test_data/0/LIDC-IDRI-0655_3/57342_000548_01-01-2000-36060_1326-NLST TLC VOL B30F-53512_doctor_anon_session_0.png]

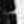

Supplement: Supplementary file 4 — Supplementary Data 1 [file 41467_2024_44946_MOESM4_ESM.zip › Supplementary Data 1/LIDC/LIDC/0/test_data/0/LIDC-IDRI-0655_4/57348_000001_01-01-2000-36060_1326-NLST TLC VOL B30F-53512_doctor_anon_session_0.png]

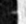

Supplement: Supplementary file 4 — Supplementary Data 1 [file 41467_2024_44946_MOESM4_ESM.zip › Supplementary Data 1/LIDC/LIDC/0/test_data/0/LIDC-IDRI-0655_4/57348_000095_01-01-2000-36060_1326-NLST TLC VOL B30F-53512_doctor_anon_session_0.png]

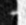

Supplement: Supplementary file 4 — Supplementary Data 1 [file 41467_2024_44946_MOESM4_ESM.zip › Supplementary Data 1/LIDC/LIDC/0/test_data/0/LIDC-IDRI-0655_4/57348_000096_01-01-2000-36060_1326-NLST TLC VOL B30F-53512_doctor_anon_session_0.png]

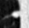

Supplement: Supplementary file 4 — Supplementary Data 1 [file 41467_2024_44946_MOESM4_ESM.zip › Supplementary Data 1/LIDC/LIDC/0/test_data/0/LIDC-IDRI-0655_4/57348_000125_01-01-2000-36060_1326-NLST TLC VOL B30F-53512_doctor_anon_session_0.png]

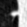

Supplement: Supplementary file 4 — Supplementary Data 1 [file 41467_2024_44946_MOESM4_ESM.zip › Supplementary Data 1/LIDC/LIDC/0/test_data/0/LIDC-IDRI-0655_4/57348_000245_01-01-2000-36060_1326-NLST TLC VOL B30F-53512_doctor_anon_session_0.png]

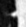

Supplement: Supplementary file 4 — Supplementary Data 1 [file 41467_2024_44946_MOESM4_ESM.zip › Supplementary Data 1/LIDC/LIDC/0/test_data/0/LIDC-IDRI-0655_4/57348_000247_01-01-2000-36060_1326-NLST TLC VOL B30F-53512_doctor_anon_session_0.png]

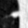

Supplement: Supplementary file 4 — Supplementary Data 1 [file 41467_2024_44946_MOESM4_ESM.zip › Supplementary Data 1/LIDC/LIDC/0/test_data/0/LIDC-IDRI-0655_4/57348_000407_01-01-2000-36060_1326-NLST TLC VOL B30F-53512_doctor_anon_session_0.png]

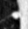

Supplement: Supplementary file 4 — Supplementary Data 1 [file 41467_2024_44946_MOESM4_ESM.zip › Supplementary Data 1/LIDC/LIDC/0/test_data/0/LIDC-IDRI-0655_4/57348_000498_01-01-2000-36060_1326-NLST TLC VOL B30F-53512_doctor_anon_session_0.png]

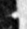

Supplement: Supplementary file 4 — Supplementary Data 1 [file 41467_2024_44946_MOESM4_ESM.zip › Supplementary Data 1/LIDC/LIDC/0/test_data/0/LIDC-IDRI-0655_4/57348_000520_01-01-2000-36060_1326-NLST TLC VOL B30F-53512_doctor_anon_session_0.png]

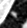

Supplement: Supplementary file 4 — Supplementary Data 1 [file 41467_2024_44946_MOESM4_ESM.zip › Supplementary Data 1/LIDC/LIDC/0/test_data/0/LIDC-IDRI-0655_5/57355_000077_01-01-2000-36060_1326-NLST TLC VOL B30F-53512_doctor_anon_session_0.png]

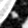

Supplement: Supplementary file 4 — Supplementary Data 1 [file 41467_2024_44946_MOESM4_ESM.zip › Supplementary Data 1/LIDC/LIDC/0/test_data/0/LIDC-IDRI-0655_5/57355_000194_01-01-2000-36060_1326-NLST TLC VOL B30F-53512_doctor_anon_session_0.png]

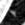

Supplement: Supplementary file 4 — Supplementary Data 1 [file 41467_2024_44946_MOESM4_ESM.zip › Supplementary Data 1/LIDC/LIDC/0/test_data/0/LIDC-IDRI-0655_5/57355_000314_01-01-2000-36060_1326-NLST TLC VOL B30F-53512_doctor_anon_session_0.png]

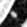

Supplement: Supplementary file 4 — Supplementary Data 1 [file 41467_2024_44946_MOESM4_ESM.zip › Supplementary Data 1/LIDC/LIDC/0/test_data/0/LIDC-IDRI-0655_5/57355_000347_01-01-2000-36060_1326-NLST TLC VOL B30F-53512_doctor_anon_session_0.png]

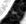

Supplement: Supplementary file 4 — Supplementary Data 1 [file 41467_2024_44946_MOESM4_ESM.zip › Supplementary Data 1/LIDC/LIDC/0/test_data/0/LIDC-IDRI-0655_5/57355_000362_01-01-2000-36060_1326-NLST TLC VOL B30F-53512_doctor_anon_session_0.png]

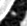

Supplement: Supplementary file 4 — Supplementary Data 1 [file 41467_2024_44946_MOESM4_ESM.zip › Supplementary Data 1/LIDC/LIDC/0/test_data/0/LIDC-IDRI-0655_5/57355_000478_01-01-2000-36060_1326-NLST TLC VOL B30F-53512_doctor_anon_session_0.png]

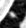

Supplement: Supplementary file 4 — Supplementary Data 1 [file 41467_2024_44946_MOESM4_ESM.zip › Supplementary Data 1/LIDC/LIDC/0/test_data/0/LIDC-IDRI-0655_5/57355_000522_01-01-2000-36060_1326-NLST TLC VOL B30F-53512_doctor_anon_session_0.png]

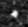

Supplement: Supplementary file 4 — Supplementary Data 1 [file 41467_2024_44946_MOESM4_ESM.zip › Supplementary Data 1/LIDC/LIDC/0/test_data/0/LIDC-IDRI-0655_6/20_000004_01-01-2000-36060_1326-NLST TLC VOL B30F-53512_doctor_anon_session_3.png]

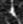

Supplement: Supplementary file 4 — Supplementary Data 1 [file 41467_2024_44946_MOESM4_ESM.zip › Supplementary Data 1/LIDC/LIDC/0/test_data/0/LIDC-IDRI-0655_6/20_000009_01-01-2000-36060_1326-NLST TLC VOL B30F-53512_doctor_anon_session_3.png]

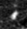

Supplement: Supplementary file 4 — Supplementary Data 1 [file 41467_2024_44946_MOESM4_ESM.zip › Supplementary Data 1/LIDC/LIDC/0/test_data/0/LIDC-IDRI-0655_6/20_000025_01-01-2000-36060_1326-NLST TLC VOL B30F-53512_doctor_anon_session_3.png]

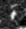

Supplement: Supplementary file 4 — Supplementary Data 1 [file 41467_2024_44946_MOESM4_ESM.zip › Supplementary Data 1/LIDC/LIDC/0/test_data/0/LIDC-IDRI-0655_6/20_000041_01-01-2000-36060_1326-NLST TLC VOL B30F-53512_doctor_anon_session_3.png]

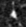

Supplement: Supplementary file 4 — Supplementary Data 1 [file 41467_2024_44946_MOESM4_ESM.zip › Supplementary Data 1/LIDC/LIDC/0/test_data/0/LIDC-IDRI-0655_6/20_000069_01-01-2000-36060_1326-NLST TLC VOL B30F-53512_doctor_anon_session_3.png]

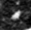

Supplement: Supplementary file 4 — Supplementary Data 1 [file 41467_2024_44946_MOESM4_ESM.zip › Supplementary Data 1/LIDC/LIDC/0/test_data/0/LIDC-IDRI-0655_6/20_000228_01-01-2000-36060_1326-NLST TLC VOL B30F-53512_doctor_anon_session_3.png]

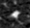

Supplement: Supplementary file 4 — Supplementary Data 1 [file 41467_2024_44946_MOESM4_ESM.zip › Supplementary Data 1/LIDC/LIDC/0/test_data/0/LIDC-IDRI-0655_6/20_000232_01-01-2000-36060_1326-NLST TLC VOL B30F-53512_doctor_anon_session_3.png]

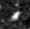

Supplement: Supplementary file 4 — Supplementary Data 1 [file 41467_2024_44946_MOESM4_ESM.zip › Supplementary Data 1/LIDC/LIDC/0/test_data/0/LIDC-IDRI-0655_6/20_000312_01-01-2000-36060_1326-NLST TLC VOL B30F-53512_doctor_anon_session_3.png]

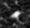

Supplement: Supplementary file 4 — Supplementary Data 1 [file 41467_2024_44946_MOESM4_ESM.zip › Supplementary Data 1/LIDC/LIDC/0/test_data/0/LIDC-IDRI-0655_6/20_000393_01-01-2000-36060_1326-NLST TLC VOL B30F-53512_doctor_anon_session_3.png]

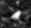

Supplement: Supplementary file 4 — Supplementary Data 1 [file 41467_2024_44946_MOESM4_ESM.zip › Supplementary Data 1/LIDC/LIDC/0/test_data/0/LIDC-IDRI-0655_6/20_000424_01-01-2000-36060_1326-NLST TLC VOL B30F-53512_doctor_anon_session_3.png]

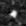

Supplement: Supplementary file 4 — Supplementary Data 1 [file 41467_2024_44946_MOESM4_ESM.zip › Supplementary Data 1/LIDC/LIDC/0/test_data/0/LIDC-IDRI-0655_6/57353_000004_01-01-2000-36060_1326-NLST TLC VOL B30F-53512_doctor_anon_session_0.png]

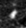

Supplement: Supplementary file 4 — Supplementary Data 1 [file 41467_2024_44946_MOESM4_ESM.zip › Supplementary Data 1/LIDC/LIDC/0/test_data/0/LIDC-IDRI-0655_6/57353_000025_01-01-2000-36060_1326-NLST TLC VOL B30F-53512_doctor_anon_session_0.png]

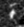

Supplement: Supplementary file 4 — Supplementary Data 1 [file 41467_2024_44946_MOESM4_ESM.zip › Supplementary Data 1/LIDC/LIDC/0/test_data/0/LIDC-IDRI-0655_6/57353_000041_01-01-2000-36060_1326-NLST TLC VOL B30F-53512_doctor_anon_session_0.png]

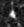

Supplement: Supplementary file 4 — Supplementary Data 1 [file 41467_2024_44946_MOESM4_ESM.zip › Supplementary Data 1/LIDC/LIDC/0/test_data/0/LIDC-IDRI-0655_6/57353_000069_01-01-2000-36060_1326-NLST TLC VOL B30F-53512_doctor_anon_session_0.png]

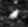

Supplement: Supplementary file 4 — Supplementary Data 1 [file 41467_2024_44946_MOESM4_ESM.zip › Supplementary Data 1/LIDC/LIDC/0/test_data/0/LIDC-IDRI-0655_6/57353_000228_01-01-2000-36060_1326-NLST TLC VOL B30F-53512_doctor_anon_session_0.png]

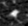

Supplement: Supplementary file 4 — Supplementary Data 1 [file 41467_2024_44946_MOESM4_ESM.zip › Supplementary Data 1/LIDC/LIDC/0/test_data/0/LIDC-IDRI-0655_6/57353_000232_01-01-2000-36060_1326-NLST TLC VOL B30F-53512_doctor_anon_session_0.png]

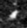

Supplement: Supplementary file 4 — Supplementary Data 1 [file 41467_2024_44946_MOESM4_ESM.zip › Supplementary Data 1/LIDC/LIDC/0/test_data/0/LIDC-IDRI-0655_6/57353_000312_01-01-2000-36060_1326-NLST TLC VOL B30F-53512_doctor_anon_session_0.png]

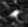

Supplement: Supplementary file 4 — Supplementary Data 1 [file 41467_2024_44946_MOESM4_ESM.zip › Supplementary Data 1/LIDC/LIDC/0/test_data/0/LIDC-IDRI-0655_6/57353_000393_01-01-2000-36060_1326-NLST TLC VOL B30F-53512_doctor_anon_session_0.png]

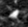

Supplement: Supplementary file 4 — Supplementary Data 1 [file 41467_2024_44946_MOESM4_ESM.zip › Supplementary Data 1/LIDC/LIDC/0/test_data/0/LIDC-IDRI-0655_6/57353_000424_01-01-2000-36060_1326-NLST TLC VOL B30F-53512_doctor_anon_session_0.png]

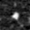

Supplement: Supplementary file 4 — Supplementary Data 1 [file 41467_2024_44946_MOESM4_ESM.zip › Supplementary Data 1/LIDC/LIDC/0/test_data/0/LIDC-IDRI-0655_6/79959_000004_01-01-2000-36060_1326-NLST TLC VOL B30F-53512_doctor_anon_session_1.png]

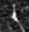

Supplement: Supplementary file 4 — Supplementary Data 1 [file 41467_2024_44946_MOESM4_ESM.zip › Supplementary Data 1/LIDC/LIDC/0/test_data/0/LIDC-IDRI-0655_6/79959_000009_01-01-2000-36060_1326-NLST TLC VOL B30F-53512_doctor_anon_session_1.png]

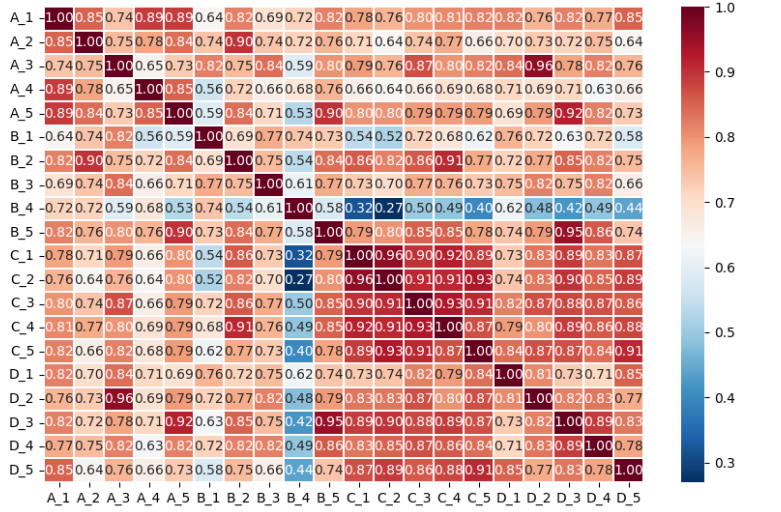

Supplement: Supplementary file 5 — Supplementary Code [file 41467_2024_44946_MOESM5_ESM.zip › Supplementary Code/RFLM_code_file/Federated learning/Analysis of robustness/Example.png]
