# Supplementary figures and images for: Robustly federated learning model for identifying high-risk patients with postoperative gastric cancer recurrence (part 1 of 2)
Source: Nat Commun. 2024 Jan 25;15:742. doi: 10.1038/s41467-024-44946-4 (PMC10811238; doi:10.1038/s41467-024-44946-4)

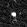

Supplement: Supplementary file 4 — Supplementary Data 1 [file 41467_2024_44946_MOESM4_ESM.zip › Supplementary Data 1/LIDC/LIDC/0/test_data/0/LIDC-IDRI-0649_1/91921_000039_01-01-2000-CT LUNG SCREEN-00055_09630_doctor_anon_session_2.png]

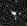

Supplement: Supplementary file 4 — Supplementary Data 1 [file 41467_2024_44946_MOESM4_ESM.zip › Supplementary Data 1/LIDC/LIDC/0/test_data/0/LIDC-IDRI-0649_1/91921_000148_01-01-2000-CT LUNG SCREEN-00055_09630_doctor_anon_session_2.png]

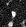

Supplement: Supplementary file 4 — Supplementary Data 1 [file 41467_2024_44946_MOESM4_ESM.zip › Supplementary Data 1/LIDC/LIDC/0/test_data/0/LIDC-IDRI-0649_10/2246_000032_01-01-2000-CT LUNG SCREEN-00055_09630_doctor_anon_session_0.png]

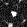

Supplement: Supplementary file 4 — Supplementary Data 1 [file 41467_2024_44946_MOESM4_ESM.zip › Supplementary Data 1/LIDC/LIDC/0/test_data/0/LIDC-IDRI-0649_10/2246_000033_01-01-2000-CT LUNG SCREEN-00055_09630_doctor_anon_session_0.png]

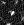

Supplement: Supplementary file 4 — Supplementary Data 1 [file 41467_2024_44946_MOESM4_ESM.zip › Supplementary Data 1/LIDC/LIDC/0/test_data/0/LIDC-IDRI-0649_10/2246_000234_01-01-2000-CT LUNG SCREEN-00055_09630_doctor_anon_session_0.png]

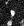

Supplement: Supplementary file 4 — Supplementary Data 1 [file 41467_2024_44946_MOESM4_ESM.zip › Supplementary Data 1/LIDC/LIDC/0/test_data/0/LIDC-IDRI-0649_10/5407_000032_01-01-2000-CT LUNG SCREEN-00055_09630_doctor_anon_session_1.png]

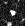

Supplement: Supplementary file 4 — Supplementary Data 1 [file 41467_2024_44946_MOESM4_ESM.zip › Supplementary Data 1/LIDC/LIDC/0/test_data/0/LIDC-IDRI-0649_10/5407_000033_01-01-2000-CT LUNG SCREEN-00055_09630_doctor_anon_session_1.png]

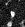

Supplement: Supplementary file 4 — Supplementary Data 1 [file 41467_2024_44946_MOESM4_ESM.zip › Supplementary Data 1/LIDC/LIDC/0/test_data/0/LIDC-IDRI-0649_10/91916_000032_01-01-2000-CT LUNG SCREEN-00055_09630_doctor_anon_session_2.png]

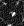

Supplement: Supplementary file 4 — Supplementary Data 1 [file 41467_2024_44946_MOESM4_ESM.zip › Supplementary Data 1/LIDC/LIDC/0/test_data/0/LIDC-IDRI-0649_10/91916_000234_01-01-2000-CT LUNG SCREEN-00055_09630_doctor_anon_session_2.png]

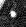

Supplement: Supplementary file 4 — Supplementary Data 1 [file 41467_2024_44946_MOESM4_ESM.zip › Supplementary Data 1/LIDC/LIDC/0/test_data/0/LIDC-IDRI-0649_2/0173_000022_01-01-2000-CT LUNG SCREEN-00055_09630_doctor_anon_session_0.png]

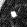

Supplement: Supplementary file 4 — Supplementary Data 1 [file 41467_2024_44946_MOESM4_ESM.zip › Supplementary Data 1/LIDC/LIDC/0/test_data/0/LIDC-IDRI-0649_2/0173_000178_01-01-2000-CT LUNG SCREEN-00055_09630_doctor_anon_session_0.png]

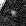

Supplement: Supplementary file 4 — Supplementary Data 1 [file 41467_2024_44946_MOESM4_ESM.zip › Supplementary Data 1/LIDC/LIDC/0/test_data/0/LIDC-IDRI-0649_2/0173_000259_01-01-2000-CT LUNG SCREEN-00055_09630_doctor_anon_session_0.png]

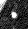

Supplement: Supplementary file 4 — Supplementary Data 1 [file 41467_2024_44946_MOESM4_ESM.zip › Supplementary Data 1/LIDC/LIDC/0/test_data/0/LIDC-IDRI-0649_2/5400_000022_01-01-2000-CT LUNG SCREEN-00055_09630_doctor_anon_session_1.png]

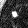

Supplement: Supplementary file 4 — Supplementary Data 1 [file 41467_2024_44946_MOESM4_ESM.zip › Supplementary Data 1/LIDC/LIDC/0/test_data/0/LIDC-IDRI-0649_2/5400_000178_01-01-2000-CT LUNG SCREEN-00055_09630_doctor_anon_session_1.png]

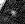

Supplement: Supplementary file 4 — Supplementary Data 1 [file 41467_2024_44946_MOESM4_ESM.zip › Supplementary Data 1/LIDC/LIDC/0/test_data/0/LIDC-IDRI-0649_2/5400_000259_01-01-2000-CT LUNG SCREEN-00055_09630_doctor_anon_session_1.png]

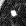

Supplement: Supplementary file 4 — Supplementary Data 1 [file 41467_2024_44946_MOESM4_ESM.zip › Supplementary Data 1/LIDC/LIDC/0/test_data/0/LIDC-IDRI-0649_2/91913_000178_01-01-2000-CT LUNG SCREEN-00055_09630_doctor_anon_session_2.png]

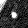

Supplement: Supplementary file 4 — Supplementary Data 1 [file 41467_2024_44946_MOESM4_ESM.zip › Supplementary Data 1/LIDC/LIDC/0/test_data/0/LIDC-IDRI-0649_2/Nodule 001_000022_01-01-2000-CT LUNG SCREEN-00055_09630_doctor_anon_session_3.png]

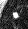

Supplement: Supplementary file 4 — Supplementary Data 1 [file 41467_2024_44946_MOESM4_ESM.zip › Supplementary Data 1/LIDC/LIDC/0/test_data/0/LIDC-IDRI-0649_2/Nodule 001_000178_01-01-2000-CT LUNG SCREEN-00055_09630_doctor_anon_session_3.png]

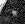

Supplement: Supplementary file 4 — Supplementary Data 1 [file 41467_2024_44946_MOESM4_ESM.zip › Supplementary Data 1/LIDC/LIDC/0/test_data/0/LIDC-IDRI-0649_2/Nodule 001_000259_01-01-2000-CT LUNG SCREEN-00055_09630_doctor_anon_session_3.png]

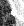

Supplement: Supplementary file 4 — Supplementary Data 1 [file 41467_2024_44946_MOESM4_ESM.zip › Supplementary Data 1/LIDC/LIDC/0/test_data/0/LIDC-IDRI-0649_3/0167_000012_01-01-2000-CT LUNG SCREEN-00055_09630_doctor_anon_session_0.png]

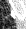

Supplement: Supplementary file 4 — Supplementary Data 1 [file 41467_2024_44946_MOESM4_ESM.zip › Supplementary Data 1/LIDC/LIDC/0/test_data/0/LIDC-IDRI-0649_3/0167_000023_01-01-2000-CT LUNG SCREEN-00055_09630_doctor_anon_session_0.png]

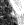

Supplement: Supplementary file 4 — Supplementary Data 1 [file 41467_2024_44946_MOESM4_ESM.zip › Supplementary Data 1/LIDC/LIDC/0/test_data/0/LIDC-IDRI-0649_3/0167_000028_01-01-2000-CT LUNG SCREEN-00055_09630_doctor_anon_session_0.png]

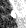

Supplement: Supplementary file 4 — Supplementary Data 1 [file 41467_2024_44946_MOESM4_ESM.zip › Supplementary Data 1/LIDC/LIDC/0/test_data/0/LIDC-IDRI-0649_3/0167_000184_01-01-2000-CT LUNG SCREEN-00055_09630_doctor_anon_session_0.png]

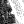

Supplement: Supplementary file 4 — Supplementary Data 1 [file 41467_2024_44946_MOESM4_ESM.zip › Supplementary Data 1/LIDC/LIDC/0/test_data/0/LIDC-IDRI-0649_3/0167_000250_01-01-2000-CT LUNG SCREEN-00055_09630_doctor_anon_session_0.png]

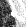

Supplement: Supplementary file 4 — Supplementary Data 1 [file 41467_2024_44946_MOESM4_ESM.zip › Supplementary Data 1/LIDC/LIDC/0/test_data/0/LIDC-IDRI-0649_3/5394_000012_01-01-2000-CT LUNG SCREEN-00055_09630_doctor_anon_session_1.png]

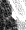

Supplement: Supplementary file 4 — Supplementary Data 1 [file 41467_2024_44946_MOESM4_ESM.zip › Supplementary Data 1/LIDC/LIDC/0/test_data/0/LIDC-IDRI-0649_3/5394_000023_01-01-2000-CT LUNG SCREEN-00055_09630_doctor_anon_session_1.png]

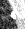

Supplement: Supplementary file 4 — Supplementary Data 1 [file 41467_2024_44946_MOESM4_ESM.zip › Supplementary Data 1/LIDC/LIDC/0/test_data/0/LIDC-IDRI-0649_3/5394_000184_01-01-2000-CT LUNG SCREEN-00055_09630_doctor_anon_session_1.png]

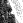

Supplement: Supplementary file 4 — Supplementary Data 1 [file 41467_2024_44946_MOESM4_ESM.zip › Supplementary Data 1/LIDC/LIDC/0/test_data/0/LIDC-IDRI-0649_3/5394_000250_01-01-2000-CT LUNG SCREEN-00055_09630_doctor_anon_session_1.png]

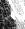

Supplement: Supplementary file 4 — Supplementary Data 1 [file 41467_2024_44946_MOESM4_ESM.zip › Supplementary Data 1/LIDC/LIDC/0/test_data/0/LIDC-IDRI-0649_3/85625_000023_01-01-2000-CT LUNG SCREEN-00055_09630_doctor_anon_session_2.png]

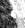

Supplement: Supplementary file 4 — Supplementary Data 1 [file 41467_2024_44946_MOESM4_ESM.zip › Supplementary Data 1/LIDC/LIDC/0/test_data/0/LIDC-IDRI-0649_3/85625_000184_01-01-2000-CT LUNG SCREEN-00055_09630_doctor_anon_session_2.png]

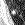

Supplement: Supplementary file 4 — Supplementary Data 1 [file 41467_2024_44946_MOESM4_ESM.zip › Supplementary Data 1/LIDC/LIDC/0/test_data/0/LIDC-IDRI-0649_4/0170_000007_01-01-2000-CT LUNG SCREEN-00055_09630_doctor_anon_session_0.png]

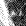

Supplement: Supplementary file 4 — Supplementary Data 1 [file 41467_2024_44946_MOESM4_ESM.zip › Supplementary Data 1/LIDC/LIDC/0/test_data/0/LIDC-IDRI-0649_4/0170_000033_01-01-2000-CT LUNG SCREEN-00055_09630_doctor_anon_session_0.png]

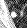

Supplement: Supplementary file 4 — Supplementary Data 1 [file 41467_2024_44946_MOESM4_ESM.zip › Supplementary Data 1/LIDC/LIDC/0/test_data/0/LIDC-IDRI-0649_4/0170_000234_01-01-2000-CT LUNG SCREEN-00055_09630_doctor_anon_session_0.png]

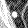

Supplement: Supplementary file 4 — Supplementary Data 1 [file 41467_2024_44946_MOESM4_ESM.zip › Supplementary Data 1/LIDC/LIDC/0/test_data/0/LIDC-IDRI-0649_4/0170_000243_01-01-2000-CT LUNG SCREEN-00055_09630_doctor_anon_session_0.png]

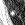

Supplement: Supplementary file 4 — Supplementary Data 1 [file 41467_2024_44946_MOESM4_ESM.zip › Supplementary Data 1/LIDC/LIDC/0/test_data/0/LIDC-IDRI-0649_4/5397_000007_01-01-2000-CT LUNG SCREEN-00055_09630_doctor_anon_session_1.png]

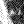

Supplement: Supplementary file 4 — Supplementary Data 1 [file 41467_2024_44946_MOESM4_ESM.zip › Supplementary Data 1/LIDC/LIDC/0/test_data/0/LIDC-IDRI-0649_4/5397_000033_01-01-2000-CT LUNG SCREEN-00055_09630_doctor_anon_session_1.png]

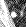

Supplement: Supplementary file 4 — Supplementary Data 1 [file 41467_2024_44946_MOESM4_ESM.zip › Supplementary Data 1/LIDC/LIDC/0/test_data/0/LIDC-IDRI-0649_4/5397_000234_01-01-2000-CT LUNG SCREEN-00055_09630_doctor_anon_session_1.png]

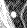

Supplement: Supplementary file 4 — Supplementary Data 1 [file 41467_2024_44946_MOESM4_ESM.zip › Supplementary Data 1/LIDC/LIDC/0/test_data/0/LIDC-IDRI-0649_4/5397_000243_01-01-2000-CT LUNG SCREEN-00055_09630_doctor_anon_session_1.png]

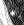

Supplement: Supplementary file 4 — Supplementary Data 1 [file 41467_2024_44946_MOESM4_ESM.zip › Supplementary Data 1/LIDC/LIDC/0/test_data/0/LIDC-IDRI-0649_4/91912_000007_01-01-2000-CT LUNG SCREEN-00055_09630_doctor_anon_session_2.png]

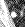

Supplement: Supplementary file 4 — Supplementary Data 1 [file 41467_2024_44946_MOESM4_ESM.zip › Supplementary Data 1/LIDC/LIDC/0/test_data/0/LIDC-IDRI-0649_4/91912_000234_01-01-2000-CT LUNG SCREEN-00055_09630_doctor_anon_session_2.png]

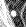

Supplement: Supplementary file 4 — Supplementary Data 1 [file 41467_2024_44946_MOESM4_ESM.zip › Supplementary Data 1/LIDC/LIDC/0/test_data/0/LIDC-IDRI-0649_4/91912_000243_01-01-2000-CT LUNG SCREEN-00055_09630_doctor_anon_session_2.png]

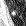

Supplement: Supplementary file 4 — Supplementary Data 1 [file 41467_2024_44946_MOESM4_ESM.zip › Supplementary Data 1/LIDC/LIDC/0/test_data/0/LIDC-IDRI-0649_4/Nodule 005_000007_01-01-2000-CT LUNG SCREEN-00055_09630_doctor_anon_session_3.png]

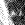

Supplement: Supplementary file 4 — Supplementary Data 1 [file 41467_2024_44946_MOESM4_ESM.zip › Supplementary Data 1/LIDC/LIDC/0/test_data/0/LIDC-IDRI-0649_4/Nodule 005_000033_01-01-2000-CT LUNG SCREEN-00055_09630_doctor_anon_session_3.png]

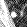

Supplement: Supplementary file 4 — Supplementary Data 1 [file 41467_2024_44946_MOESM4_ESM.zip › Supplementary Data 1/LIDC/LIDC/0/test_data/0/LIDC-IDRI-0649_4/Nodule 005_000234_01-01-2000-CT LUNG SCREEN-00055_09630_doctor_anon_session_3.png]

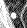

Supplement: Supplementary file 4 — Supplementary Data 1 [file 41467_2024_44946_MOESM4_ESM.zip › Supplementary Data 1/LIDC/LIDC/0/test_data/0/LIDC-IDRI-0649_4/Nodule 005_000243_01-01-2000-CT LUNG SCREEN-00055_09630_doctor_anon_session_3.png]

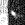

Supplement: Supplementary file 4 — Supplementary Data 1 [file 41467_2024_44946_MOESM4_ESM.zip › Supplementary Data 1/LIDC/LIDC/0/test_data/0/LIDC-IDRI-0649_5/0182_000027_01-01-2000-CT LUNG SCREEN-00055_09630_doctor_anon_session_0.png]

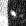

Supplement: Supplementary file 4 — Supplementary Data 1 [file 41467_2024_44946_MOESM4_ESM.zip › Supplementary Data 1/LIDC/LIDC/0/test_data/0/LIDC-IDRI-0649_5/0182_000227_01-01-2000-CT LUNG SCREEN-00055_09630_doctor_anon_session_0.png]

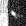

Supplement: Supplementary file 4 — Supplementary Data 1 [file 41467_2024_44946_MOESM4_ESM.zip › Supplementary Data 1/LIDC/LIDC/0/test_data/0/LIDC-IDRI-0649_5/91918_000027_01-01-2000-CT LUNG SCREEN-00055_09630_doctor_anon_session_2.png]

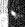

Supplement: Supplementary file 4 — Supplementary Data 1 [file 41467_2024_44946_MOESM4_ESM.zip › Supplementary Data 1/LIDC/LIDC/0/test_data/0/LIDC-IDRI-0649_5/Nodule 013_000027_01-01-2000-CT LUNG SCREEN-00055_09630_doctor_anon_session_3.png]

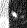

Supplement: Supplementary file 4 — Supplementary Data 1 [file 41467_2024_44946_MOESM4_ESM.zip › Supplementary Data 1/LIDC/LIDC/0/test_data/0/LIDC-IDRI-0649_5/Nodule 013_000227_01-01-2000-CT LUNG SCREEN-00055_09630_doctor_anon_session_3.png]

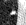

Supplement: Supplementary file 4 — Supplementary Data 1 [file 41467_2024_44946_MOESM4_ESM.zip › Supplementary Data 1/LIDC/LIDC/0/test_data/0/LIDC-IDRI-0649_6/0183_000082_01-01-2000-CT LUNG SCREEN-00055_09630_doctor_anon_session_0.png]

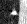

Supplement: Supplementary file 4 — Supplementary Data 1 [file 41467_2024_44946_MOESM4_ESM.zip › Supplementary Data 1/LIDC/LIDC/0/test_data/0/LIDC-IDRI-0649_6/0183_000254_01-01-2000-CT LUNG SCREEN-00055_09630_doctor_anon_session_0.png]

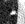

Supplement: Supplementary file 4 — Supplementary Data 1 [file 41467_2024_44946_MOESM4_ESM.zip › Supplementary Data 1/LIDC/LIDC/0/test_data/0/LIDC-IDRI-0649_6/5412_000082_01-01-2000-CT LUNG SCREEN-00055_09630_doctor_anon_session_1.png]

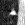

Supplement: Supplementary file 4 — Supplementary Data 1 [file 41467_2024_44946_MOESM4_ESM.zip › Supplementary Data 1/LIDC/LIDC/0/test_data/0/LIDC-IDRI-0649_6/5412_000254_01-01-2000-CT LUNG SCREEN-00055_09630_doctor_anon_session_1.png]

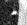

Supplement: Supplementary file 4 — Supplementary Data 1 [file 41467_2024_44946_MOESM4_ESM.zip › Supplementary Data 1/LIDC/LIDC/0/test_data/0/LIDC-IDRI-0649_6/91919_000082_01-01-2000-CT LUNG SCREEN-00055_09630_doctor_anon_session_2.png]

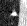

Supplement: Supplementary file 4 — Supplementary Data 1 [file 41467_2024_44946_MOESM4_ESM.zip › Supplementary Data 1/LIDC/LIDC/0/test_data/0/LIDC-IDRI-0649_6/91919_000254_01-01-2000-CT LUNG SCREEN-00055_09630_doctor_anon_session_2.png]

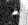

Supplement: Supplementary file 4 — Supplementary Data 1 [file 41467_2024_44946_MOESM4_ESM.zip › Supplementary Data 1/LIDC/LIDC/0/test_data/0/LIDC-IDRI-0649_6/Nodule 012_000082_01-01-2000-CT LUNG SCREEN-00055_09630_doctor_anon_session_3.png]

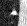

Supplement: Supplementary file 4 — Supplementary Data 1 [file 41467_2024_44946_MOESM4_ESM.zip › Supplementary Data 1/LIDC/LIDC/0/test_data/0/LIDC-IDRI-0649_6/Nodule 012_000254_01-01-2000-CT LUNG SCREEN-00055_09630_doctor_anon_session_3.png]

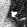

Supplement: Supplementary file 4 — Supplementary Data 1 [file 41467_2024_44946_MOESM4_ESM.zip › Supplementary Data 1/LIDC/LIDC/0/test_data/0/LIDC-IDRI-0649_7/2248_000113_01-01-2000-CT LUNG SCREEN-00055_09630_doctor_anon_session_0.png]

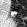

Supplement: Supplementary file 4 — Supplementary Data 1 [file 41467_2024_44946_MOESM4_ESM.zip › Supplementary Data 1/LIDC/LIDC/0/test_data/0/LIDC-IDRI-0649_7/2248_000168_01-01-2000-CT LUNG SCREEN-00055_09630_doctor_anon_session_0.png]

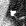

Supplement: Supplementary file 4 — Supplementary Data 1 [file 41467_2024_44946_MOESM4_ESM.zip › Supplementary Data 1/LIDC/LIDC/0/test_data/0/LIDC-IDRI-0649_7/5408_000113_01-01-2000-CT LUNG SCREEN-00055_09630_doctor_anon_session_1.png]

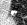

Supplement: Supplementary file 4 — Supplementary Data 1 [file 41467_2024_44946_MOESM4_ESM.zip › Supplementary Data 1/LIDC/LIDC/0/test_data/0/LIDC-IDRI-0649_7/5408_000168_01-01-2000-CT LUNG SCREEN-00055_09630_doctor_anon_session_1.png]

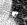

Supplement: Supplementary file 4 — Supplementary Data 1 [file 41467_2024_44946_MOESM4_ESM.zip › Supplementary Data 1/LIDC/LIDC/0/test_data/0/LIDC-IDRI-0649_7/91917_000168_01-01-2000-CT LUNG SCREEN-00055_09630_doctor_anon_session_2.png]

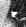

Supplement: Supplementary file 4 — Supplementary Data 1 [file 41467_2024_44946_MOESM4_ESM.zip › Supplementary Data 1/LIDC/LIDC/0/test_data/0/LIDC-IDRI-0649_7/Nodule 006_000113_01-01-2000-CT LUNG SCREEN-00055_09630_doctor_anon_session_3.png]

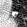

Supplement: Supplementary file 4 — Supplementary Data 1 [file 41467_2024_44946_MOESM4_ESM.zip › Supplementary Data 1/LIDC/LIDC/0/test_data/0/LIDC-IDRI-0649_7/Nodule 006_000168_01-01-2000-CT LUNG SCREEN-00055_09630_doctor_anon_session_3.png]

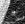

Supplement: Supplementary file 4 — Supplementary Data 1 [file 41467_2024_44946_MOESM4_ESM.zip › Supplementary Data 1/LIDC/LIDC/0/test_data/0/LIDC-IDRI-0649_7/Nodule 006_000244_01-01-2000-CT LUNG SCREEN-00055_09630_doctor_anon_session_3.png]

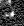

Supplement: Supplementary file 4 — Supplementary Data 1 [file 41467_2024_44946_MOESM4_ESM.zip › Supplementary Data 1/LIDC/LIDC/0/test_data/0/LIDC-IDRI-0649_8/0178_000032_01-01-2000-CT LUNG SCREEN-00055_09630_doctor_anon_session_0.png]

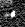

Supplement: Supplementary file 4 — Supplementary Data 1 [file 41467_2024_44946_MOESM4_ESM.zip › Supplementary Data 1/LIDC/LIDC/0/test_data/0/LIDC-IDRI-0649_8/0178_000033_01-01-2000-CT LUNG SCREEN-00055_09630_doctor_anon_session_0.png]

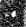

Supplement: Supplementary file 4 — Supplementary Data 1 [file 41467_2024_44946_MOESM4_ESM.zip › Supplementary Data 1/LIDC/LIDC/0/test_data/0/LIDC-IDRI-0649_8/0178_000234_01-01-2000-CT LUNG SCREEN-00055_09630_doctor_anon_session_0.png]

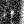

Supplement: Supplementary file 4 — Supplementary Data 1 [file 41467_2024_44946_MOESM4_ESM.zip › Supplementary Data 1/LIDC/LIDC/0/test_data/0/LIDC-IDRI-0649_8/0178_000243_01-01-2000-CT LUNG SCREEN-00055_09630_doctor_anon_session_0.png]

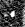

Supplement: Supplementary file 4 — Supplementary Data 1 [file 41467_2024_44946_MOESM4_ESM.zip › Supplementary Data 1/LIDC/LIDC/0/test_data/0/LIDC-IDRI-0649_8/5405_000234_01-01-2000-CT LUNG SCREEN-00055_09630_doctor_anon_session_1.png]

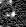

Supplement: Supplementary file 4 — Supplementary Data 1 [file 41467_2024_44946_MOESM4_ESM.zip › Supplementary Data 1/LIDC/LIDC/0/test_data/0/LIDC-IDRI-0649_8/91915_000032_01-01-2000-CT LUNG SCREEN-00055_09630_doctor_anon_session_2.png]

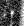

Supplement: Supplementary file 4 — Supplementary Data 1 [file 41467_2024_44946_MOESM4_ESM.zip › Supplementary Data 1/LIDC/LIDC/0/test_data/0/LIDC-IDRI-0649_8/91915_000243_01-01-2000-CT LUNG SCREEN-00055_09630_doctor_anon_session_2.png]

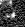

Supplement: Supplementary file 4 — Supplementary Data 1 [file 41467_2024_44946_MOESM4_ESM.zip › Supplementary Data 1/LIDC/LIDC/0/test_data/0/LIDC-IDRI-0649_8/Nodule 011_000032_01-01-2000-CT LUNG SCREEN-00055_09630_doctor_anon_session_3.png]

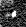

Supplement: Supplementary file 4 — Supplementary Data 1 [file 41467_2024_44946_MOESM4_ESM.zip › Supplementary Data 1/LIDC/LIDC/0/test_data/0/LIDC-IDRI-0649_8/Nodule 011_000033_01-01-2000-CT LUNG SCREEN-00055_09630_doctor_anon_session_3.png]

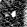

Supplement: Supplementary file 4 — Supplementary Data 1 [file 41467_2024_44946_MOESM4_ESM.zip › Supplementary Data 1/LIDC/LIDC/0/test_data/0/LIDC-IDRI-0649_8/Nodule 011_000234_01-01-2000-CT LUNG SCREEN-00055_09630_doctor_anon_session_3.png]

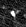

Supplement: Supplementary file 4 — Supplementary Data 1 [file 41467_2024_44946_MOESM4_ESM.zip › Supplementary Data 1/LIDC/LIDC/0/test_data/0/LIDC-IDRI-0649_9/0174_000013_01-01-2000-CT LUNG SCREEN-00055_09630_doctor_anon_session_0.png]

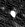

Supplement: Supplementary file 4 — Supplementary Data 1 [file 41467_2024_44946_MOESM4_ESM.zip › Supplementary Data 1/LIDC/LIDC/0/test_data/0/LIDC-IDRI-0649_9/0174_000050_01-01-2000-CT LUNG SCREEN-00055_09630_doctor_anon_session_0.png]

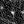

Supplement: Supplementary file 4 — Supplementary Data 1 [file 41467_2024_44946_MOESM4_ESM.zip › Supplementary Data 1/LIDC/LIDC/0/test_data/0/LIDC-IDRI-0649_9/0174_000194_01-01-2000-CT LUNG SCREEN-00055_09630_doctor_anon_session_0.png]

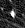

Supplement: Supplementary file 4 — Supplementary Data 1 [file 41467_2024_44946_MOESM4_ESM.zip › Supplementary Data 1/LIDC/LIDC/0/test_data/0/LIDC-IDRI-0649_9/0174_000224_01-01-2000-CT LUNG SCREEN-00055_09630_doctor_anon_session_0.png]

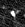

Supplement: Supplementary file 4 — Supplementary Data 1 [file 41467_2024_44946_MOESM4_ESM.zip › Supplementary Data 1/LIDC/LIDC/0/test_data/0/LIDC-IDRI-0649_9/5404_000013_01-01-2000-CT LUNG SCREEN-00055_09630_doctor_anon_session_1.png]

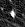

Supplement: Supplementary file 4 — Supplementary Data 1 [file 41467_2024_44946_MOESM4_ESM.zip › Supplementary Data 1/LIDC/LIDC/0/test_data/0/LIDC-IDRI-0649_9/5404_000224_01-01-2000-CT LUNG SCREEN-00055_09630_doctor_anon_session_1.png]

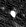

Supplement: Supplementary file 4 — Supplementary Data 1 [file 41467_2024_44946_MOESM4_ESM.zip › Supplementary Data 1/LIDC/LIDC/0/test_data/0/LIDC-IDRI-0649_9/91914_000050_01-01-2000-CT LUNG SCREEN-00055_09630_doctor_anon_session_2.png]

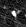

Supplement: Supplementary file 4 — Supplementary Data 1 [file 41467_2024_44946_MOESM4_ESM.zip › Supplementary Data 1/LIDC/LIDC/0/test_data/0/LIDC-IDRI-0649_9/Nodule 002_000013_01-01-2000-CT LUNG SCREEN-00055_09630_doctor_anon_session_3.png]

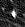

Supplement: Supplementary file 4 — Supplementary Data 1 [file 41467_2024_44946_MOESM4_ESM.zip › Supplementary Data 1/LIDC/LIDC/0/test_data/0/LIDC-IDRI-0649_9/Nodule 002_000224_01-01-2000-CT LUNG SCREEN-00055_09630_doctor_anon_session_3.png]

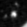

Supplement: Supplementary file 4 — Supplementary Data 1 [file 41467_2024_44946_MOESM4_ESM.zip › Supplementary Data 1/LIDC/LIDC/0/test_data/0/LIDC-IDRI-0651_1/1_000057_01-01-2000-00685_30041-30421_doctor_anon_session_3.png]

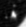

Supplement: Supplementary file 4 — Supplementary Data 1 [file 41467_2024_44946_MOESM4_ESM.zip › Supplementary Data 1/LIDC/LIDC/0/test_data/0/LIDC-IDRI-0651_1/1_000112_01-01-2000-00685_30041-30421_doctor_anon_session_3.png]

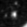

Supplement: Supplementary file 4 — Supplementary Data 1 [file 41467_2024_44946_MOESM4_ESM.zip › Supplementary Data 1/LIDC/LIDC/0/test_data/0/LIDC-IDRI-0651_1/1_000117_01-01-2000-00685_30041-30421_doctor_anon_session_3.png]

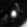

Supplement: Supplementary file 4 — Supplementary Data 1 [file 41467_2024_44946_MOESM4_ESM.zip › Supplementary Data 1/LIDC/LIDC/0/test_data/0/LIDC-IDRI-0651_1/1_000202_01-01-2000-00685_30041-30421_doctor_anon_session_3.png]

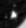

Supplement: Supplementary file 4 — Supplementary Data 1 [file 41467_2024_44946_MOESM4_ESM.zip › Supplementary Data 1/LIDC/LIDC/0/test_data/0/LIDC-IDRI-0651_1/1_000216_01-01-2000-00685_30041-30421_doctor_anon_session_3.png]

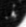

Supplement: Supplementary file 4 — Supplementary Data 1 [file 41467_2024_44946_MOESM4_ESM.zip › Supplementary Data 1/LIDC/LIDC/0/test_data/0/LIDC-IDRI-0651_1/1_000242_01-01-2000-00685_30041-30421_doctor_anon_session_3.png]

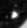

Supplement: Supplementary file 4 — Supplementary Data 1 [file 41467_2024_44946_MOESM4_ESM.zip › Supplementary Data 1/LIDC/LIDC/0/test_data/0/LIDC-IDRI-0651_1/1_000317_01-01-2000-00685_30041-30421_doctor_anon_session_3.png]

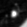

Supplement: Supplementary file 4 — Supplementary Data 1 [file 41467_2024_44946_MOESM4_ESM.zip › Supplementary Data 1/LIDC/LIDC/0/test_data/0/LIDC-IDRI-0651_1/1_000394_01-01-2000-00685_30041-30421_doctor_anon_session_3.png]

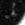

Supplement: Supplementary file 4 — Supplementary Data 1 [file 41467_2024_44946_MOESM4_ESM.zip › Supplementary Data 1/LIDC/LIDC/0/test_data/0/LIDC-IDRI-0651_1/1_000420_01-01-2000-00685_30041-30421_doctor_anon_session_3.png]

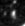

Supplement: Supplementary file 4 — Supplementary Data 1 [file 41467_2024_44946_MOESM4_ESM.zip › Supplementary Data 1/LIDC/LIDC/0/test_data/0/LIDC-IDRI-0651_1/1_000523_01-01-2000-00685_30041-30421_doctor_anon_session_3.png]

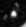

Supplement: Supplementary file 4 — Supplementary Data 1 [file 41467_2024_44946_MOESM4_ESM.zip › Supplementary Data 1/LIDC/LIDC/0/test_data/0/LIDC-IDRI-0651_1/1_000527_01-01-2000-00685_30041-30421_doctor_anon_session_3.png]

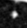

Supplement: Supplementary file 4 — Supplementary Data 1 [file 41467_2024_44946_MOESM4_ESM.zip › Supplementary Data 1/LIDC/LIDC/0/test_data/0/LIDC-IDRI-0655_1/57346_000018_01-01-2000-36060_1326-NLST TLC VOL B30F-53512_doctor_anon_session_0.png]

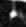

Supplement: Supplementary file 4 — Supplementary Data 1 [file 41467_2024_44946_MOESM4_ESM.zip › Supplementary Data 1/LIDC/LIDC/0/test_data/0/LIDC-IDRI-0655_1/57346_000117_01-01-2000-36060_1326-NLST TLC VOL B30F-53512_doctor_anon_session_0.png]

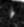

Supplement: Supplementary file 4 — Supplementary Data 1 [file 41467_2024_44946_MOESM4_ESM.zip › Supplementary Data 1/LIDC/LIDC/0/test_data/0/LIDC-IDRI-0655_1/57346_000246_01-01-2000-36060_1326-NLST TLC VOL B30F-53512_doctor_anon_session_0.png]

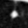

Supplement: Supplementary file 4 — Supplementary Data 1 [file 41467_2024_44946_MOESM4_ESM.zip › Supplementary Data 1/LIDC/LIDC/0/test_data/0/LIDC-IDRI-0655_1/57346_000373_01-01-2000-36060_1326-NLST TLC VOL B30F-53512_doctor_anon_session_0.png]
