# Supplementary material for: Robustly federated learning model for identifying high-risk patients with postoperative gastric cancer recurrence
Source: Nat Commun. 2024 Jan 25;15:742. doi: 10.1038/s41467-024-44946-4 (PMC10811238; doi:10.1038/s41467-024-44946-4)
Supplement: Supplementary file 6 — Reporting Summary [file 41467_2024_44946_MOESM6_ESM.pdf]

nature portfolio

Corresponding author(s): Wansheng Long

Last updated by author(s): Dec 20, 2023

Reporting Summary

Nature Portfolio wishes to improve the reproducibility of the work that we publish. This form provides structure for consistency and transparency in reporting. For further information on Nature Portfolio policies, see our Editorial Policies and the Editorial Policy Checklist.

Statistics

For all statistical analyses, confirm that the following items are present in the figure legend, table legend, main text, or Methods section.

n/a	Confirmed	
☐	☒	The exact sample size (n) for each experimental group/condition, given as a discrete number and unit of measurement
☐	☒	A statement on whether measurements were taken from distinct samples or whether the same sample was measured repeatedly
☐	☒	The statistical test(s) used AND whether they are one- or two-sided<br>*Only common tests should be described solely by name; describe more complex techniques in the Methods section.*
☒	☐	A description of all covariates tested
☒	☐	A description of any assumptions or corrections, such as tests of normality and adjustment for multiple comparisons
☐	☒	A full description of the statistical parameters including central tendency (e.g. means) or other basic estimates (e.g. regression coefficient) AND variation (e.g. standard deviation) or associated estimates of uncertainty (e.g. confidence intervals)
☐	☒	For null hypothesis testing, the test statistic (e.g. F, t, r) with confidence intervals, effect sizes, degrees of freedom and P value noted<br>*Give P values as exact values whenever suitable.*
☒	☐	For Bayesian analysis, information on the choice of priors and Markov chain Monte Carlo settings
☐	☒	For hierarchical and complex designs, identification of the appropriate level for tests and full reporting of outcomes
☐	☒	Estimates of effect sizes (e.g. Cohen's d, Pearson's r), indicating how they were calculated

Our web collection on statistics for biologists contains articles on many of the points above.

Software and code

Policy information about availability of computer code

Data collection	No software was used
Data analysis	Python (version 3.6) was used to complete the federated learning framework;<br>Matlab (version 2020b) was used for feature screening and classifier construction:<br>Pytorch (version 1.7.1-GPU) was used to build the deep learning framework.<br>Statistical tests were performed using R studio (version 3.4.0) and IBM SPSS Statistics (version 20.0).<br>The codes of the proposed method are also available at GitHub (https://github.com/baofengguat/RFLM-project/tree/master).

For manuscripts utilizing custom algorithms or software that are central to the research but not yet described in published literature, software must be made available to editors and reviewers. We strongly encourage code deposition in a community repository (e.g. GitHub). See the Nature Portfolio guidelines for submitting code & software for further information.

Data

Policy information about availability of data

All manuscripts must include a data availability statement. This statement should provide the following information, where applicable:

- Accession codes, unique identifiers, or web links for publicly available datasets
- A description of any restrictions on data availability
- For clinical datasets or third party data, please ensure that the statement adheres to our policy

Multicentre data on gastric cancer: The data that support the findings of this study are available at the web repository of "https://pan.baidu.com/s/1KgUi57fU9erapmaUVvjeXA? " and its extraction code can be obtained from the corresponding author upon a separate request.
Lung Image Database Consortium (LIDC-IDRI): https://wiki.cancerimagingarchive.net/display/Public/LIDC-IDRI

Research involving human participants, their data, or biological material

Policy information about studies with human participants or human data. See also policy information about sex, gender (identity/presentation), and sexual orientation and race, ethnicity and racism.

Reporting on sex and gender	In this study only the sex of the patients is considered and the distribution of patient gender is provided in the data description section. Additionally, a stratified analysis based on sex is presented in the supplementary materials to ensure that the experimental results are not influenced by gender factors.
Reporting on race, ethnicity, or other socially relevant groupings	This study does not involve reporting on race, ethnicity, or other socially relevant groupings.
Population characteristics	This study is a retrospective investigation that involved a total of 641 patients across four data centers. The average age of patients at centre A was 60 years, with males comprising 63.1% (n-181); at centre B, the average age was 62 years, with males accounting for 69.2% (n=97); at centre C, the average age was 56 years, with males making up 58.7% (n=64); and at centre D, the average age was 56 years, with males representing 55.5% (n=55).
Recruitment	This study falls under the category of retrospective research.
Ethics oversight	This study was implemented under the approval of the Jiangmen Central Hospital, Meizhou People's Hospital, The First Affiliated Hospital of Sun Yat-sen University and Dongguan People's Hospital.

Note that full information on the approval of the study protocol must also be provided in the manuscript.

Field-specific reporting

Please select the one below that is the best fit for your research. If you are not sure, read the appropriate sections before making your selection.

☒ Life sciences ☐ Behavioural & social sciences ☐ Ecological, evolutionary & environmental sciences

For a reference copy of the document with all sections, see nature.com/documents/nr-reporting-summary-flat.pdf

Life sciences study design

All studies must disclose on these points even when the disclosure is negative.

Sample size	This study adopted a retrospective data collection methodology, encompassing patients diagnosed with gastric cancer, confirmed by surgical pathology, from April 2008 to November 2019 across four medical centers. A total of 641 patients were enrolled at four medical data centres. We employed no statistical models to predetermine sample size, but our sample sizes are at the same level as or larger than those reported in previous studies with similar research aims. For example, a study on postoperative recurrence of gastric cancer published in the Annals ofl Oncology in 2021 utilized 546 patients for experimentation [1]. Similarly, another research on postoperative recurrence of gastric cancer, published in the JOURNAL OF CLINICAL ONCOLOGY in 2021, included 226 patients in the study [2].<br>[1].Park, SH, Lim, DH, Sohn, TS, et al. A randomized phase III trial comparing adjuvant single-agent S1, S-1 with oxaliplatin, and postoperativel chemoradiation with S-1 and oxaliplatin in patients with node-positive gastric cancer after D2 resection: the ARTIST 2 trial. ANN ONCOL.2020; 32 (3): 368-374. doi: 10.1016/j.annonc.2020.11.017.<br>[2].Watanabe, H, Hayashi, T, Koumori, K, et al. Impact of postoperative complications on recurrence in pathological stage II/III gastric cancerl patients who received curative resection followed by adjuvant S-1 chemotherapy. J CLIN ONCOL. 2020; 38 (4_suppl): 320-320. doi: 10.1200/jco.2020.38.4_suppl.320.
Data exclusions	Exclusion criteria for data in this study were as follows:(1) poor quality CT images where lesion visualization was unclear: (2) detection of other malignant tumors in CT scans. The primary endpoints of this study were local recurrence and non-recurrence cases, with a minimum follow-up time of five years for all other cases. Patients underwent follow-up checks every 3 to 6 months during the first 2 years, every 6 to 12 months over the following 3 years, and subsequently annually. The primary follow-up methods incorporated contrast-enhanced abdominal CT, gastroscopy, and tumor biomarker examinations, among others. Following the aforementioned screening, a total of 641 patients were included in the study.
Replication	We conducted robustness experiments, including cross-validation and interference resistance tests. All replication attempts were

successful,and multiple experimental runs yielded similar results.

Randomization: The division of the training and testing sets for the multi-centre advanced gastric cancer data in the study employed a random splitting strategy.

Blinding: The deep learning models were developed on the training cohort. All training was done without any information from the test cohort and the reader study cohort.

Reporting for specific materials, systems and methods

We require information from authors about some types of materials, experimental systems and methods used in many studies. Here, indicate whether each material, system or method listed is relevant to your study. If you are not sure if a list item applies to your research, read the appropriate section before selecting a response.

Materials & experimental systems

n/a	Involved in the study	
☒	☐	Antibodies
☒	☐	Eukaryotic cell lines
☒	☐	Palaeontology and archaeology
☒	☐	Animals and other organisms
☒	☐	Clinical data
☒	☐	Dual use research of concern
☒	☐	Plants

Methods

n/a	Involved in the study	
☒	☐	ChIP-seq
☒	☐	Flow cytometry
☒	☐	MRI-based neuroimaging

Plants

Seed stocks: This study does not involve this aspect.

Novel plant genotypes: This study does not involve this aspect.

Authentication: This study does not involve this aspect.
